# Distribution and pathogenic diversity in *Fusarium udum* Butler isolates: the causal agent of pigeonpea Fusarium wilt

**DOI:** 10.1186/s12870-022-03526-8

**Published:** 2022-03-26

**Authors:** Ravikumara B. M, Ramanagouda G, M. K. Naik, Rameshwar Telangre, Mamta Sharma

**Affiliations:** 1 Crop Protection and Seed Health, Accelerated Crop Improvement, International Crops Research Institute for the Semi-Arid Tropics (ICRISAT), Patancheru, 502 324 Telangana India; 2 Department of Plant Pathology, University of Agricultural Sciences, Raichur, 584 102 Karnataka India; 3 Precision Phenotyping for Biotic-Abiotic Stresses & Nutrition-Research Program-Asia, International Crops Research Institute for the Semi-Arid Tropics (ICRISAT), Patancheru, 502 324 Telangana India

**Keywords:** *Cajanus cajan*, *Fusarium udum*, Variability, Phenotyping, Variants

## Abstract

**Background:**

Fusarium wilt (*Fusarium udum* Butler), an important soil-borne disease of pigeonpea [*Cajanus cajan* (L.)], causes significant yield losses across the major pigeonpea production regions. Widespread and high diversity in *F. udum* hampers the breeding for pigeonpea wilt resistance. The study aimed to elucidate the pathogenic diversity and distribution of *F. udum* variants in major pigeonpea growing regions of India.

**Results:**

The roving survey was conducted in major pigeonpea-growing states of India to collect the *F. udum* isolates. Pathogenic variability of 60 *F. udum* isolates which are selected from diverse geographical locations and pathogenicity test were performed against 11 pigeonpea host differentials cultivars [ICP 8858, ICP 8859, ICP 8862, ICP 8863, ICP 9174, C 11, BDN 1, BDN 2, LRG 30, ICP 2376 and Bahar (ICP 7197)]. The current study indicated distribution of *F. udum* isolates into nine variants (0, 1, 2, 3, 4, 5, 6, 7 and 8). Variant-2 and 3 were found to be widespread and predominant in most pigeonpea producing regions. Variant-7 (Karnataka) and Variant-8 (Madhya Pradesh and Maharashtra) were found highly virulent, as most of the host differentials were susceptible to these variants. Three host differential cultivars namely ICP 9174, BDN-2 and Bahar (ICP 7197) were found resistant to most of the *F. udum* isolates.

**Conclusion:**

The present study generated significant information in terms of variants of *F. udum* which could be used further for the deployment of location-specific wilt resistant cultivars for optimized disease-management strategies. Study is also useful for development of broad-based wilt resistant cultivars to curtail the possible epidemics.

**Supplementary Information:**

The online version contains supplementary material available at 10.1186/s12870-022-03526-8.

## Background

Pigeonpea (*Cajanus cajan* (L.) Millsp.) is the most widely grown and consumed grain legume in the tropic and sub-tropic regions of the world [1]. Being a hardy crop, it is a natural choice for small and marginal farmers particularly on low-fertilizer input soils. Pigeonpea is known for its quality protein, vitamin B, carotene, and ascorbic acid [2, 3], animal feed, fuelwood, green manure and in improving soil fertility through biological nitrogen fixation [4, 5]. The crop is cultivated in more than 38 countries including India, Myanmar, Kenya, Malawi, Tanzania and Uganda which are some of the top producing countries [6].

Pigeonpea yield in most production regions is well below its potential and has been stagnated for several decades [7] mainly due to biotic and abiotic stresses, especially during critical seedling and reproductive stages [8]. Among the biotic stresses, Fusarium wilt caused by *Fusarium udum* Butler is widespread fungal disease in all pigeonpea-growing areas and causes significant yield losses [9–12]. Recent surveys indicated that Fusarium wilt incidence is increasing significantly in the major pigeonpea production regions of India [13]. Being a soil-borne pathogen, *F. udum* enters the host through the root systems, and infection starts from seedling stage but maximum expression of the disease is more prominent at flowering and podding stage [14]. Infected plants show gradual chlorosis, drooping and subsequent death of the plants under field conditions. Vascular discoloration and purple band on the stem extending upwards are the major symptoms of wilt in pigeonpea.

The disease was first recorded by Butler [15] in Bihar, India, and subsequently reported from other pigeonpea-growing countries [1, 9, 16]. In 2006, the disease was reported from Southern Africa—in Mozambique’s Zambezia province [10]. The *F. udum* isolates from the same or different geographical origin have shown high degree of cultural [13] and pathogenic variations [17–19]. The high diversity and virulence could be due to the influence of environment factors and soil inhabitant nature of *F. udum* [20–22].

Crop rotation, seed treatment with fungicides, use of biocontrol agents and resistant cultivars are the most common practices for the efficient management of Fusarium wilt [23]. Seed treatment with fungicide is not economical and fails to give complete protection. Therefore, it is imperative to understand the status of *F. udum* pathogenic variation for successful deployment of resistant cultivars in production regions [22, 24]. Existence of variability in *F. udum* has been cited as a major drawback in the development of wilt resistant cultivars [24]. Evidence suggests that most of the released commercial cultivars are showing susceptibility or differential reactions in the farmers’ fields [8, 22, 25–27]. Cultivars which are designated as resistant to *F. udum* were showing the moderate to high degree of susceptibility in some locations [24, 28].

Studies on *F. udum* diversity have been conducted with a limited number of isolates on a few pigeonpea host differential cultivars [14]. A better knowledge on pathogenic variability of the population would alleviate the efficiency of pigeonpea breeding programs of the country. Therefore, we aimed to assess the current distribution of variability of *F. udum* in major pigeonpea growing regions of India to substantiate the location-specific Fusarium wilt resistance breeding program.

## Results

### Fusarium wilt incidence

Fusarium wilt incidence in surveyed fields ranged from 0–70 per cent. Average wilt incidence was maximum in Karnataka state (11.72%) followed by Maharashtra (9.88%), Telangana (9.43%), Madhya Pradesh (7.41%) and Tamil Nadu (6.87%). Details of wilt incidence in different surveyed locations is provided in supplementary table 1a. Among the surveyed states, the commonly grown cultivars were TS 3R, BSMR 175, BSMR 736, Gulyal red, ICP 8836 (Maruti) and Karitogari in Karnataka; BSMR 736, Maruti, BDN 1, BDN 2, BDN 7, Asha and Gulyal red in Maharashtra; ICPL 87, TS 3R, LRG 30 Asha, Maruti and Abhaya in Telangana; Jagrathi, JA 4, Jawahar, Asha and Khargoan 7 in Madhya Pradesh; Vamban, C 11, Khargoan 1 and Asha in Tamil Nadu; and some local cultivars in other states (Supplementary table 1a).

### Pathogenicity of isolates

A total of 104 isolates were subjected to pathogenicity test on susceptible ICP 2376 cultivar using artificial root-dip inoculation technique (Supplementary table 1b). All the isolates were identified as pathogenic to pigeonpea. The re-isolated cultures were morphologically and microscopically similar to that of original cultures and confirmed as *F. udum.* Further, representative isolates were confirmed through sequencing of ITS region of the pathogen. The amplicon product was sequenced, and a BLASTn search revealed 100% sequence similarity to *Fusarium udum* species. The sequences were submitted to the GenBank database (accession no. MZ298786 to MZ298799). High degree of pathogenic variation among the *F. udum* isolates were observed during pathogenicity test on ICP 2376 cultivar. Incubation period (IP) (days from the inoculation to first appearance of symptoms) of isolates varied from 9 to 20 days. The IP was noticed in Fu 28 isolate at day 9 (from Raichur, Karnataka) and in Fu 65 isolate (from Parbhani, Maharashtra) at 20 days’ post incubation. The average latent period (duration from first disease appearance to complete wilting of the plant) of the isolates ranged between 4–5 days.

### Pathogenic variability using host differential cultivars

**Fig. 1:**
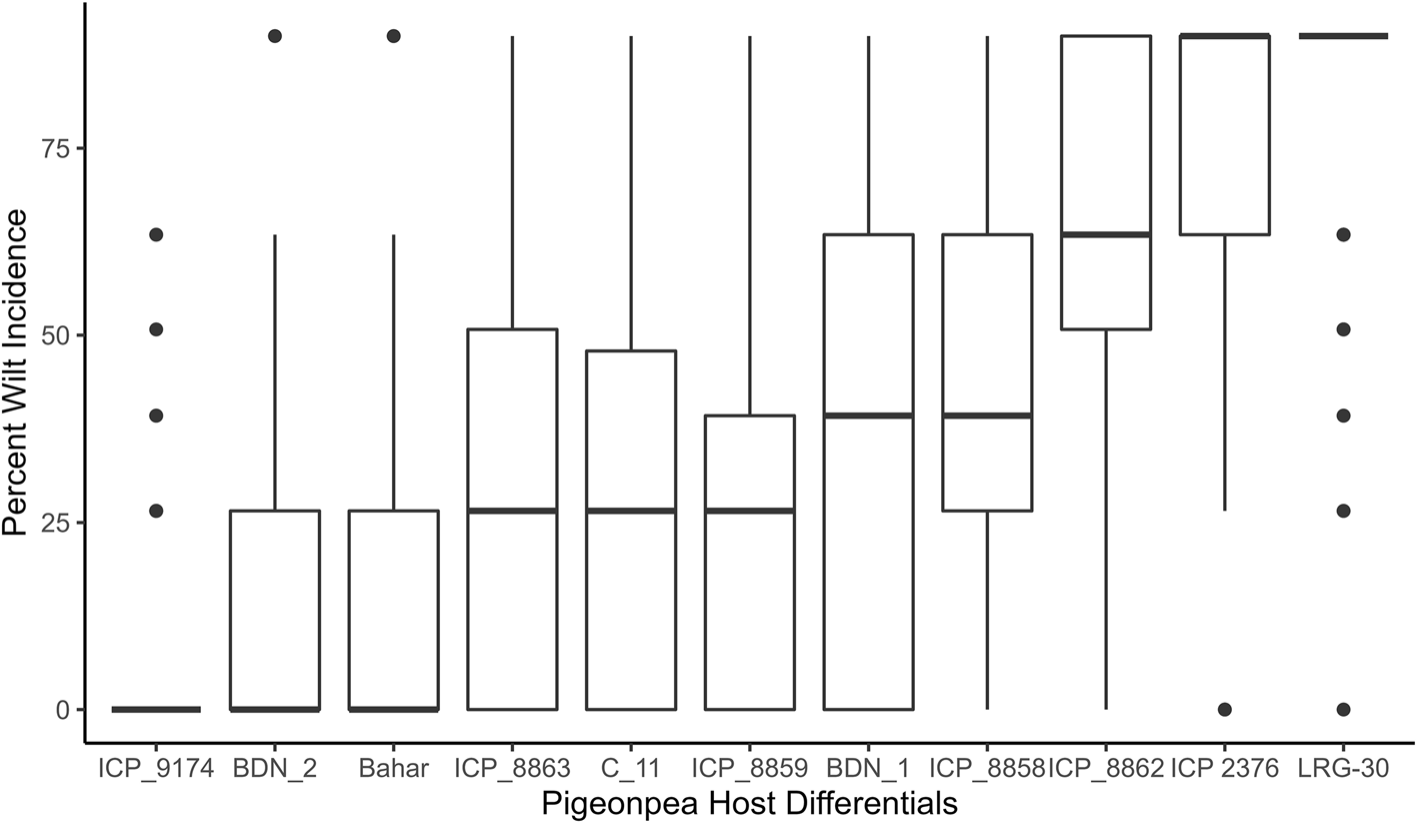
Per cent wilt incidence in pigeonpea host differential genotypes against *Fusarium udum* isolates. Box edges represents the upper and lower quantile with median value shown in the middle of the box

The ANOVA (Table 1) indicated that the per cent wilt incidence and its interactions on host differentials were significant at *p* ≤ 0.01. Irrespective of isolates, the average wilt incidence was least in cultivar ICP 9174 (4.48%) followed by BDN 2 (10.55%), Bahar (12.24%), ICP 8859 (25.17%) and C 11 (25.87%). However, maximum susceptibility to *F. udum* isolates was recorded in cultivars ICP 8863 (37.71%), BDN 1 (37.91%), ICP 8858 (44.18%), ICP 8862 (64.18%), ICP 2376 (75.12%) and LRG 30 (83.28%) (Supplementary table 2). Boxplot clearly represented the range of wilt incidence in different host differentials (Fig. 1).

**Table 1:** Analysis of variance of interaction between pigeonpea host differentials and *Fusarium udum* isolates

Source of variation	DF	Mean Square	F Value	Pr > F
Host differentials (G)	10	114,987	404.12	< 0.0001
*F. udum* isolates (I)	59	5851	20.53	< 0.0001
G * I	590	786	2.76	< 0.0001
Residuals	1500	285

**Fig. 2:**
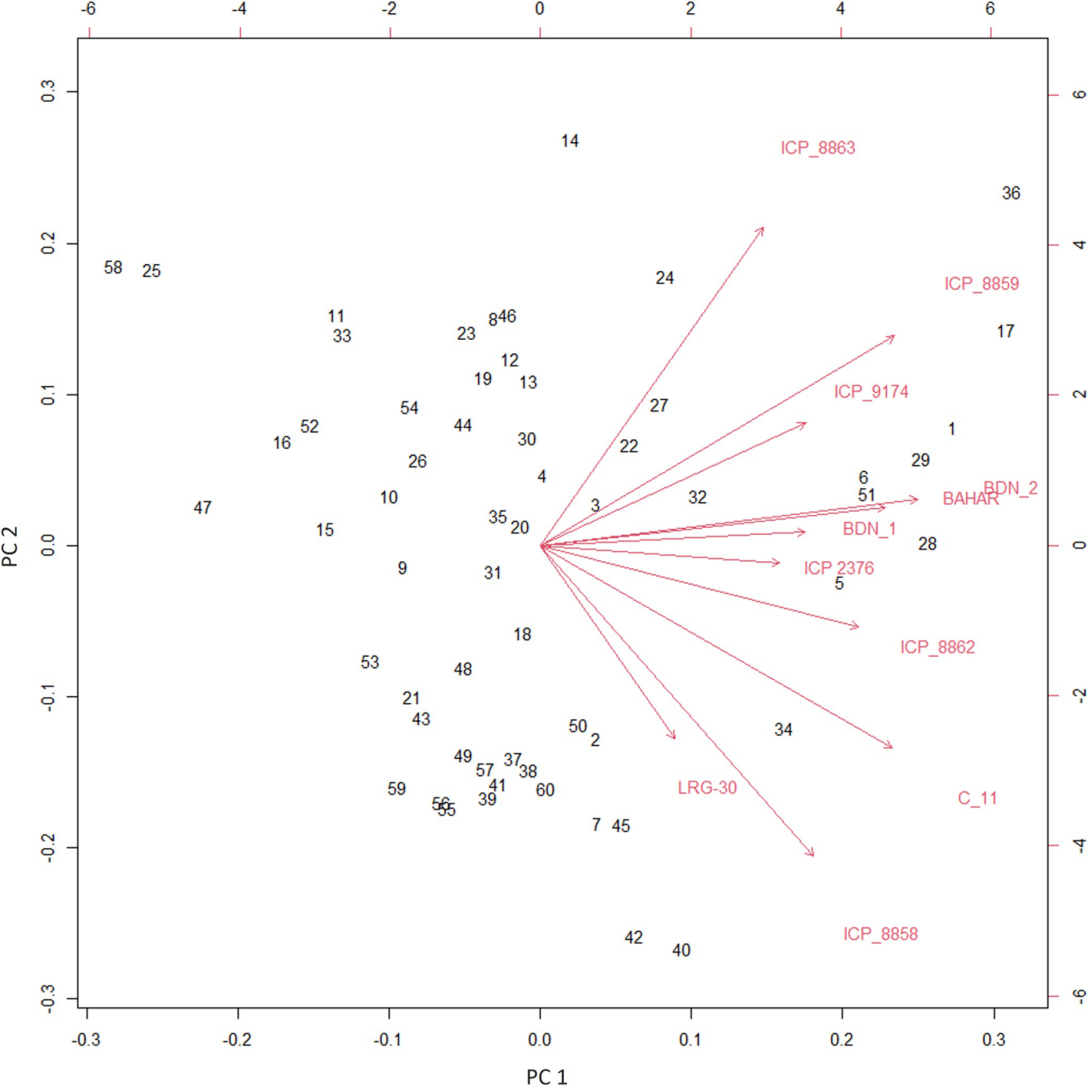
Biplot showing the first two principal axes of interaction (comp1 vs Comp2) for the wilt incidence on 11 genotypes against 60 isolates of *Fusarium udum*

The biplot analysis showed the dispersion of *F. udum* isolates on determined plane of PC1 and PC2 components. PC1 and PC2 explain 75.66% (56.52 and 19.14%) variation in wilt incidence and spread of *F. udum* isolates based on pathogenic reaction on host differentials. The vectors of variables are positively correlated with each other with clear separation of resistant and susceptible genotypes. The PC1 exhibited a positive direction for all the variables and PC2 exhibited a negative direction for susceptible genotypes. Thus, in the cartesian plane defined by components PC1 and PC2, vectors of resistant differentials viz., ICP 9174, BDN 1, BDN 2, ICP 8859, ICP 8863 and Bahar, share the upper-right quadrant of the plane. On the other hand, vectors of the susceptible differentials C 11, ICP 8862, ICP 8858, ICP 2376 and LRG 30 variables were found in the lower right quadrant. We could observe the dispersion of less-virulent isolates on upper left side of the plane and more-virulent isolates on upper right side of the quadrant of the plane. The intermediate-to-medium pathogenic isolates were found associated in both the quadrants of the PC1 and negative quadrant of PC2 (Fig. 2).

### Distribution of *Fusarium udum* variants in India

**Fig. 3:**
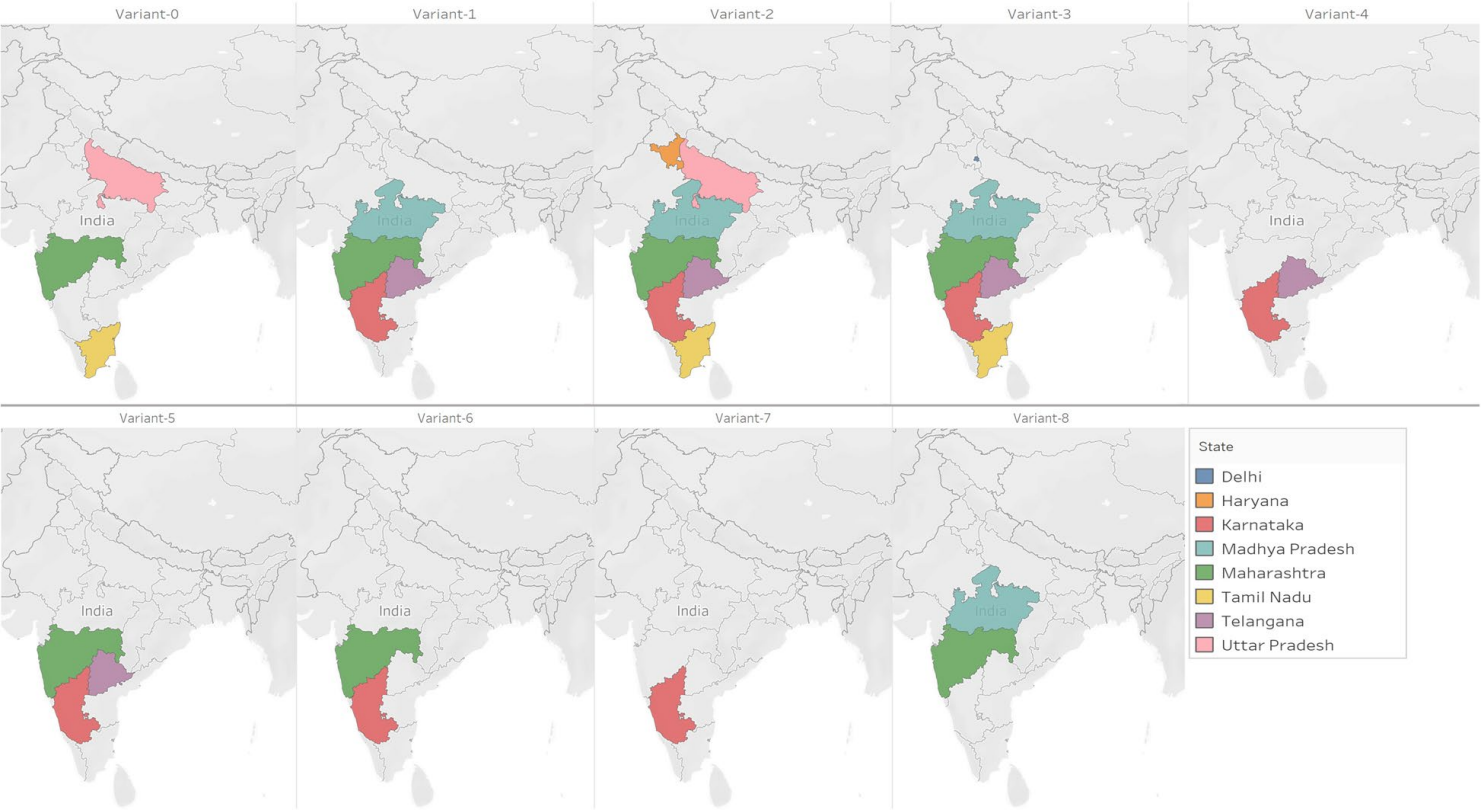
Geographical distribution of *Fusarium udum* variants in India

Disease incidence was converted into three categories as R–resistant (0–10% wilting), MR–moderately resistant (10–30% wilting) and S–susceptible (> 30% wilting) (Supplementary table 2 and Table 2). Based on the distinct pathogenic reaction of isolates on host differentials, isolates were categorized into nine variants (variant-0, variant-1, variant-2, variant-3, variant-4, variant-5, vaiant-6, variant-7 and variant-8). Details of variants and host differential reactions are summarized in Table 3. Out of 9 variants, maximum distribution of *F. udum* variants was noticed Karnataka and Maharashtra (7 variants in each state) followed by Telangana (6 variants), Madhya Pradesh (4 variants), Tamil Nadu (3 variants), Uttar Pradesh (2 variants) and one variant each in Delhi and Haryana states. Geographical distribution and prevalence of variants in major pigeonpea-growing states of India are presented in Fig. 3.

**Table 2:** Reaction of pigeonpea standard host differentials to 60 isolates of *Fusarium udum* in India

Isolates Code	Pigeonpea host differentials											Variants
	**ICP 9174**	**BDN 2**	**Bahar**	**ICP 8863**	**C 11**	**ICP 8859**	**BDN 1**	**ICP 8858**	**ICP 8862**	**ICP 2376**	**LRG 30**	
Fu 105	R	R	R	R	R	R	R	R	R	S	S	Variant-0
Fu 83	R	R	R	R	R	R	R	MR	R	S	S	Variant-0
Fu 65	R	R	MR	S	R	MR	R	MR	R	S	S	Variant-0
Fu 25	R	R	R	R	R	R	R	R	S	S	S	Variant-1
Fu 43	R	R	R	R	R	R	R	R	S	S	S	Variant-1
Fu 16	R	R	R	R	R	R	R	R	S	S	S	Variant-1
Fu 38	R	R	R	S	R	R	R	MR	S	S	S	Variant-1
Fu 98	R	R	R	S	R	R	S	R	S	S	S	Variant-1
Fu 19	R	R	R	S	R	R	S	R	S	S	S	Variant-1
Fu 100	R	R	R	S	R	R	S	MR	S	S	S	Variant-1
Fu 58	R	R	R	S	R	S	S	MR	S	S	S	Variant-1
Fu 24	R	R	R	S	R	S	S	R	S	S	S	Variant-1
Fu 106	R	R	R	R	R	R	R	S	S	S	S	Variant-2
Fu 15	R	R	R	R	R	MR	R	S	S	S	S	Variant-2
Fu 70	R	MR	R	S	R	R	R	S	S	S	S	Variant-2
Fu 27	R	R	R	S	R	MR	R	S	S	S	S	Variant-2
Fu 46	R	R	R	S	R	MR	R	S	S	S	S	Variant-2
Fu 79	R	R	R	S	R	MR	R	S	S	S	S	Variant-2
Fu 31	R	MR	R	S	R	MR	R	S	S	S	S	Variant-2
Fu 81	R	MR	MR	S	R	MR	R	S	S	S	S	Variant-2
Fu 103	R	R	R	S	S	R	R	S	S	S	S	Variant-2
Fu 101	R	MR	R	R	S	R	MR	S	S	S	S	Variant-2
Fu 74	R	R	R	R	S	MR	MR	S	S	S	S	Variant-2
Fu 104	R	R	R	R	S	MR	MR	S	S	S	S	Variant-2
Fu 77	R	R	MR	R	S	MR	MR	S	S	S	S	Variant-2
Fu 93	R	R	MR	R	S	R	MR	S	S	S	S	Variant-2
Fu 12	R	MR	MR	R	S	R	MR	S	S	S	S	Variant-2
Fu 36	R	R	R	R	R	R	S	S	S	S	S	Variant-3
Fu 78	R	R	R	R	R	R	S	S	S	S	S	Variant-3
Fu 99	R	R	R	R	R	R	S	S	S	S	S	Variant-3
Fu 34	R	R	MR	MR	R	MR	S	S	S	S	S	Variant-3
Fu 76	R	R	R	R	S	MR	S	S	S	S	S	Variant-3
Fu 4	R	R	MR	R	S	MR	S	S	S	S	S	Variant-3
Fu 86	R	R	MR	R	S	MR	S	S	S	S	S	Variant-3
Fu 107	R	R	MR	R	S	MR	S	S	S	S	S	Variant-3
Fu 72	R	R	MR	R	S	R	S	S	S	S	S	Variant-3
Fu 95	R	MR	MR	R	S	R	S	S	S	S	S	Variant-3
Fu 75	R	MR	MR	R	S	MR	S	S	S	S	S	Variant-3
Fu 29	R	MR	R	R	S	MR	S	S	S	S	S	Variant-3
Fu 60	R	MR	R	R	S	MR	S	S	S	S	S	Variant-3
Fu 73	R	MR	R	R	S	MR	S	S	S	S	S	Variant-3
Fu 80	R	MR	R	R	S	MR	S	S	S	S	S	Variant-3
Fu 8	R	R	R	S	MR	S	S	S	S	S	S	Variant-4
Fu 13	R	MR	R	S	MR	S	S	S	S	S	S	Variant-4
Fu 37	R	MR	MR	S	R	S	S	S	S	S	S	Variant-4
Fu 21	R	R	MR	S	MR	S	S	S	S	S	S	Variant-4
Fu 42	R	R	MR	S	MR	S	S	S	S	S	S	Variant-4
Fu 23	R	R	R	S	S	S	S	S	S	S	S	Variant-5
Fu 6	R	R	MR	S	S	S	S	S	S	S	S	Variant-5
Fu 61	R	R	R	S	S	S	S	S	S	S	S	Variant-5
Fu 68	R	MR	S	R	S	S	S	S	S	S	S	Variant-6
Fu 49	R	R	S	S	S	S	R	S	S	S	S	Variant-6
Fu 10	R	S	S	S	S	MR	S	S	S	S	S	Variant-7
Fu 3	R	S	S	S	S	S	S	S	S	S	S	Variant-7
Fu 11	R	S	S	S	S	S	S	S	S	S	S	Variant-7
Fu 28	R	S	S	S	S	S	S	S	S	S	S	Variant-7
Fu 54	R	S	S	S	S	S	S	S	S	S	S	Variant-7
Fu 55	S	S	MR	S	S	S	S	S	S	S	S	Variant-8
Fu 71	S	S	MR	S	S	S	S	S	S	S	S	Variant-8
Fu 97	S	S	MR	S	S	S	S	S	S	S	S	Variant-8

*R* Resistant, *MR* Moderately Resistant, *S *Susceptible

**Table 3:** Grouping of *Fusarium udum* variants based on their reaction to pigeonpea host differentials

Variants	Pigeonpea host differentials										
	**ICP 9174**	**BDN2**	**Bahar**	**ICP 8863**	**C11**	**ICP 8859**	**BDN1**	**ICP 8858**	**ICP 8862**	**ICP 2376**	**LRG 30**
Variant-0	R	R	R/MR	R/S	R	R/MR	R	R/MR	R	S	S
Variant-1	R	R	R	R/S	R	R/S	R/S	R/MR	S	S	S
Variant-2	R	R/MR	R/MR	R/S	R/S	R/MR	R/MR	S	S	S	S
Variant-3	R	R/MR	R/MR	R/S	R/S	R/MR	S	S	S	S	S
Variant-4	R	R/MR	R/MR	S	R/MR	S	S	S	S	S	S
Variant-5	R	R	R/MR	S	S	S	S	S	S	S	S
Variant-6	R	R/MR	S	R/S	S	S	R/S	S	S	S	S
Variant-7	R	S	S	S	S	MR/S	S	S	S	S	S
Variant-8	S	S	MR	S	S	S	S	S	S	S	S

*R* Resistant, *MR* Moderately Resistant, *S* Susceptible

Of all the variants, variant-2 was found to be predominantly distributed in most of the pigeonpea-growing states (Karnataka, Madhya Pradesh, Maharashtra, Tamil Nadu, Telangana and Uttar Pradesh, Haryana) followed by variant-3 (Delhi, Karnataka, Madhya Pradesh, Maharashtra, Tamil Nadu and Telangana); variant-1 (Karnataka, Madhya Pradesh, Maharashtra and Telangana); and variant-0 (Maharashtra, Tamil Nadu and Uttar Pradesh). However, variants-4, 5, and 6 were predominantly found in Karnataka, Maharashtra and Telangana. Variant 7 reported from Karnataka (Bidar); and variant-8 from Madhya Pradesh and Maharashtra states (Seoni, Solapur, Latur) was found to be most virulent as most of the host differentials were susceptible (Table 4).

**Table 4:** Geographical distribution of pigeonpea *Fusarium udum* variants in India

Variants	Delhi^a^	Haryana	Karnataka	Madhya Pradesh	Maharashtra	Tamil Nadu	Telangana	Uttar Pradesh
Variant-0					Parbhani^b^	Dharmapuri		Kanpur
Variant-1			Mandya, Kalaburagi, Bidar	Seoni, Sehore	Jalna		Warangal, Rangareddy	
Variant-2		Hissar	Bangalore North, Raichur, Kalaburagi	Sehore, Jabalpur	Akola	Vellore, Krishnagiri	Warangal	Kanpur
Variant-3	New Delhi		Chitradurga, Yadgir, Raichur	Seoni, Narashinghpur, Jabalpur	Solapur, Yavatmal	Coimbatore, Tiruvannamalai, Krishnagiri, Dharmapuri, Vellore	Mahbubnagar	
Variant-4			Kalaburagi				Mahbubnagar, Warangal, Medak	
Variant-5			Raichur		Jalna		Mahbubnagar	
Variant-6			Kalaburagi		Buldhana		Medak	
Variant-7			Bidar					
Variant-8				Seoni	Solapur, Latur			

^**a**^State names in India; ^**b**^District names in particular state

## Discussion

Pigeonpea is mainly grown in Karnataka, Maharashtra, Madhya Pradesh, Telangana, Uttar Pradesh, Tamil Nadu, Andhra Pradesh, Gujarat and Bihar states in India. To a lesser extent, it is also grown in Chhattisgarh, Rajasthan, Odisha, Punjab, Haryana and some parts of the North-Eastern states of India [29]. Fusarium wilt disease is the most prevalent in India, and quite serious in Malawi, Tanzania and Kenya [9]. Significant yield losses have been observed in many susceptible cultivars throughout the pigeonpea-growing areas [30]. The survey has shown that maximum average wilt incidence was in Karnataka, Maharashtra and Telangana states. Fusarium wilt incidence was generally more in farmer’s field with local cultivars such as Kari togari, Gulyal local and Kattibheeja as compared to cultivars with improved disease resistance. Cultivars Asha and Maruti were found to be wilt resistant in these states. Similar kind of wilt incidence was recorded in different years in these states by some researchers [8, 31–33].

*F. udum* is host specific and is pathogenic to only pigeonpea [34–36]. Wilt-affected plants showed various types of symptoms, viz., drooping of lower leaves, yellowing of leaves, interveinal chlorosis, ultimately leading to the death of the entire plant. Symptoms produced in root-dip pathogenicity experiment are in agreement with previous reports [37]. We found that all 104 *F. udum* isolates were pathogenic to susceptible cultivar (ICP 2376). The 75 pigeonpea wilted samples from 55 sites in 12 districts of Kenya and found all isolates to be pathogenic to the susceptible KAT 60/8 variety [18]. In a similar method, 32 isolates of *F. udum* which were collected from 21 districts of seven states of India were also pathogenic to susceptible pigeonpea cultivar [26].

Virulence being a quantitative measure of pathogenicity denotes the severity of the disease caused by a pathogen on a particular host [38]. A few *F. udum* isolates were moderate to highly pathogenic to resistant C-11 and Muktha cultivars [39]. The five *F. udum* isolates collected from Warangal, Khammam and Ranga Reddy districts of Telangana varied greatly in virulence, disease incidence, disease reaction, latent period and virulence index on the set of seven host differentials and three locally grown cultivars [40]. In our study, we observed variable reactions of 60 *F. udum* isolates on 11 host differentials. In our study, we observed variable reactions of 60 *F. udum* isolates on 11 host differentials. Wilt incidence was more in LRG 30, ICP 2376, ICP 8862, ICP 8858, BDN 1 and ICP 8863 cultivars as compared to ICP 9174, BDN 2, Bahar, C 11 and ICP 8859. Pathogenic variability in *F. udum* isolates varied among host differentials and isolates from different geographical locations. For instance, pigeonpea line ICP 9145, which was wilt resistant at Katumani (Kenya), ICRISAT-Patancheru (India) and Malawi, but was highly susceptible (71% wilt) at Kiboko (Kenya) [20]. Similar observations were reported using 18 pigeonpea differentials against seven isolates of *F. udum* from India [31] and by using six pigeonpea differentials against 12 isolates from Kenya [24]. In India, prevalence of *F. udum* races identified by using four pigeonpea lines against 11 *F. udum* isolates [41]. Variability in wilt reactions within locations, within states or between countries could be due to the existence of different virulent forms of isolates as well as interactions with type of cultivars and environment [20, 21]. In general, Fusarium species and populations are more prone to boom-and-bust type cycle, crop, cropping systems and practices in the field leading to regular selection pressure for greater fitness or variation in the virulence [44].

Characterization of variability in *F. udum* isolates is essential for the development and deployment of efficient resistant cultivars in major pigeonpea-growing areas of India. In the present study, *F. udum* isolates were divided into nine different variants of India. These variants were distributed widely across all the major pigeonpea-producing regions of India. Previously, three distinct *F. udum* pathogenic groups were identified based on the reactions on four pigeonpea lines [41], and five pathogenic variants (variants 1, 2, 3, 4 and 5) in India based on reactions to ICP 8863, ICP 9174, C 11, Bahar and LRG 30 (Dhar et al., 2011). Wide distribution of variants clearly indicates the need for the development of resistant cultivars and their deployment. Variants-2 and 3 were predominately distributed among the major pigeonpea-growing states. Further, presence of more virulent variants (variants-7 and 8) were reported from Karnataka and Maharashtra states. Emergence of more virulent forms of *F. udum* could be attributed to mono-cropping or mixed cropping of pigeonpea creating continuous availability of host and favourable environmental conditions. On the contrary, in previous studies, it is reported the distribution of single pathogenic groups/variants of *F. udum* in different locations, viz., strain 1 from Gwalior and Akola, strain 2 from Dholi, Varanasi, Bangalore and Kanpur, and strain 3 from Patancheru, Rahuri and Kalaburagi districts of India [42]. It is assumed that the movement of planting material along with pathogen is the main reason for the distribution of pathogen variants. Commercial and most widely grown cultivar ICP 8863, which is popular for over three decades in southern and central parts of India [43], found to be susceptible to the highly virulent *F. udum* variants 7 and 8.

## Conclusions

This is the first comprehensive study elucidating *F. udum* pathogenic diversity and distribution in major pigeonpea-producing regions in India. The study clearly indicated the differential reaction (resistant/susceptible) of isolates on host differentials. Three cultivars, ICP 9174, BDN-2 and to some extent Bahar (ICP 7197), have resistant reaction to wilt over diverse geographies and can be used as resistant donor in pigeonpea wilt-resistant breeding programs. The existence of more than one variant in a state has been witnessed indicating a significant impact of isolate x genotype x environment interactions. Wide distribution of *F. udum* variants clearly indicates the need for the development and deployment of stable and broad-based wilt resistant cultivars in the major pigeonpea producing regions.

## Methods

### Collection and maintenance of isolates

An intensive field roving surveys were conducted *Kharif* 2013–14 and *Kharif* 2014–15 at flowering to maturity stage targeting major pigeonpea growing regions of India (Supplementary table 1a & b). Total 1191 fields were surveyed randomly at every 15–20 km and in each surveyed field, three 1 × 1 m quadrat were inspected along a diagonal transect. The number of plants per quadrat was counted and plants with typical wilt symptoms were also counted. The average wilt incidence was calculated using the below formula.

$$\mathrm{Per}\;\mathrm{cent}\;\mathrm{wilt}\;\mathrm{incidence}\;=\frac{\mathrm{Number}\;\mathrm{of}\;\mathrm{plants}\;\mathrm{wilted}}{\mathrm{Total}\;\mathrm{number}\;\mathrm{of}\;\mathrm{plants}\;\mathrm{observed}}\mathrm X100.$$

Symptomatic plants with brown discoloration of vascular tissues were selected for pathogen isolation. Roots of the infected plants were cut into small pieces (0.5 cm^2^) and surface- sterilized with 1% (v/v) sodium hypochlorite solution for 15–30 s. These pieces were washed thoroughly in sterile distilled water thrice, aseptically transferred to Petri plates containing Potato Dextrose Agar (PDA) (200 g sliced potato, 20 g dextrose, 20 g agar and l L of water) media and incubated at 25 ± 1 ºC in a 12 h light/dark for 36–48 h. A total 104 isolates were isolated from the surveyed fields. All the isolates were subjected to single-spore isolation on 2% (w/v) water agar and single germinating conidia were transferred to fresh PDA after 12–24 h. Cultures were maintained in refrigerated conditions (4 ºC) for future use and one-time sub-cultured isolates (original culture) were used for pathogenicity test and pathogenic variability studies.

### Inoculum preparation and pathogenicity test

The pathogenicity assay was conducted for all 104 isolates by inoculating the pathogen on a susceptible cultivar (ICP 2376) using root dip inoculation technique [37]. Seedlings were raised in polythene covers filled with 2/3 volume-sterilized river-sand under greenhouse condition at 25 ± 2 ºC. Seeds were surface-sterilized before sowing using 2% (v/v) sodium hypochlorite for 2 min, and 25 to 30 seeds were sown in each polythene covers and allowed to grow for eight days. Seedlings were carefully uprooted and roots were washed under running tap water to remove sand particles. Roots of the seedlings were dipped in the fungal spore suspension (6 × 10^6^ spores/ml) for two minutes. For mass production of spore inoculum, a 7-mm disc of actively growing *F. udum* culture from each isolate was put separately into a 250 ml conical flask containing 100 ml of sterilized potato dextrose broth and incubated for 7 days in an incubator shaker at 25 ± 1 °C with 125 rpm. Inoculated seedlings were transplanted into 12-cm pre-irrigated pots containing sterilized black soil and sand (3:1). Five seedlings were transplanted per pot in three replications. Un-inoculated control was included where root tips were dipped in sterile distilled water and transplanted into the pots. Plants were checked every two days for the appearance of wilt symptoms up to 60 days after inoculation [26]. The pathogen was re-isolated from all the isolates and compared with original cultures to prove the Koch’s postulates.

All 104 isolates were identified as *F. udum* based on the pathogenicity test and microscopic colony characters as described [34, 35, 44]. Further, molecular identification of the pathogen based on the internal transcribed spacer (ITS) region was conducted, wherein the polymerase chain reaction (PCR) amplification of few representative isolates (Fu 25, Fu 111, Fu 27, Fu 109, Fu 103, Fu 56, Fu 6, Fu 13, Fu 44, Fu 102, Fu 87, Fu 92, Fu 104 and Fu 79) was carried out using ITS1 (forward) and ITS4 (reverse) primers [13].

### Host differentials and pathogenic variability

Based on pathogenicity test and geographical representation of isolates in major pigeonpea-production regions of India, 60 isolates were selected for pathogenic variability study on host differentials. Standard eleven pigeonpea host differentials viz., ICP 8858, ICP 8859, ICP 8862, ICP 8863, ICP 9174, C 11, BDN 1, BDN 2, LRG 30, ICP 2376 and Bahar (ICP 7197) which were shown consistent differential reactions to *F. udum* at several locations in India were selected for this study [26, 45–48] (Table 5). Seeds of these genotypes / cultivars were obtained from ICRISAT pigeonpea-breeding program and RS Paroda, Genebank at Patancheru, India. ICRISAT has the world’s largest repositories of genetic resources of its mandate crops, and at present conserves more than 120,000 accessions from 144 countries (https://www.icrisat.org/gene-bank/). The genetic purity of the seed was maintained by selfing each genotype under protected condition.

**Table 5:** Details of pigeonpea host differential genotypes used in virulence profiling studies against *Fusarium udum* isolates

Genotype	Alternate accession	Origin	Province	Biological status	Growth habit	Flower color/ streak/density	Flowering (d)	Maturity (d)	Pod color	Seed color	100 seed wt (g)
Bahar	P 1258; NP 65; ICP 7197	India	New Delhi	Advanced/ Improved cv	Semis-spreading	Yellow	148	225	Mixed (G & P)	Orange	9.5
BDN 1	ICP 7182	India	Maharashtra	Advanced/ Improved cv	Semi-spreading	Yellow–red-few	116	175	Green	Dark brown	9.7
BDN 2	ICP 7623	India	Maharashtra	Advanced/ Improved cv	Semi-spreading	Yellow–red-few	106	154	Mixed (G & P)	Cream	8.8
C 11	ICP 7118	India	Maharashtra	Advanced/ Improved cv	Semi-spreading	Yellow–red-few	116	165	Green	Orange	10.3
ICP 2376	RG 102; P 3888	India	Telangana	Breeding/Research material	Spreading	Yellow–red-dense	110	150	Green	White	9.2
ICP 8858	IC-WR 1; 1-W6-W3XB	India	Telangana	Breeding/ Research material	Semi-spreading	Yellow–red-few	133	182	Mixed (G & P)	Orange	10.8
ICP 8859	IC-WR 2; 6443-W14XB	India	Telangana	Breeding/ Research material	Compact	Yellow–red-few	159	240	Mixed (G & P)	White	7.1
ICP 8862	IC-WR 5; 7119-S251X-W15X	India	Telangana	Breeding/ Research material	Compact	Yellow–red-plain	148	196	Mixed (G & P)	White	16.8
ICP 8863	IC-WR 6; 7626-W1X-W16XB; Maruti	India	Telangana	Advanced/ Improved cv	Semi-spreading	Yellow–red-few	111	158	Mixed (G & P)	Orange	9.5
ICP 9174	JM 2467	Kenya	Eastern Kisasi	Traditional cv./ Landrace	Compact	Ivory	165	252	Mixed (G & P)	White	13.9
LRG 30	ICP 8518	India	Telangana	Advanced/ Improved cv	Semi-spreading	Light yellow	137	189	Mixed (G & P)	Orange	7.0

^*^G & P Green & Purple pod color, *d* Days, *g* Gram

**Fig. 4:**
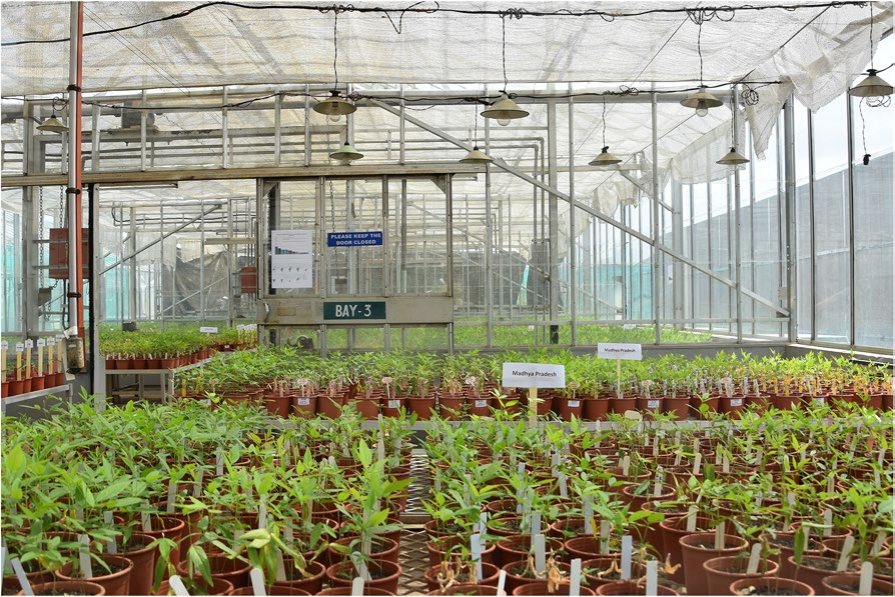
Overview of virulence profiling of pigeonpea *Fusarium udum* under greenhouse conditions at ICRISAT, India

The interaction of *F. udum* isolates on host differential cultivars was tested by following the above mentioned root-dip inoculation technique in completely randomized design (CRD) under controlled environment conditions in greenhouse at 25 ± 2 ºC (Fig. 4). Experiment was conducted in three replicates with five plants in each treatment. Data on wilt incidence was recorded at 15 day’s intervals up to 90 days after inoculation. After 90 days, per cent disease incidence was calculated by using the formula mentioned above. Based on the disease incidence, host differentials were categorized as resistant (0–10% wilt incidence), moderately resistant (10–30% wilt incidence) and susceptible (> 30.1% wilt incidences) [24, 26].

### Data curation and analysis

Cumulative Fusarium wilt incidence in each location was calculated by considering the average per cent wilt incidence in different fields of particular location. Data on pathogenic variability was analysed by analysis of variance (ANOVA) to compare the difference between the host differentials against isolates using SAS 9.4 (Statistical Analysis Systems Institute Inc. 2016). Prior to analysis, the per cent wilt incidence data was subjected to arcsine-transformation to make residuals normal and then back-transformed for the presentation of the results [49, 50]. Significance of mean differences within host differentials and isolates was tested by the Student’s t-test in combination with Bonferroni correction at *P* = 0.01 level of probability.

Boxplots were generated using R statistical program (R Development Core Team 2020) to visualize the distribution pattern of per cent wilt incidence of different *F. udum* isolates on pigeonpea host differentials. The interaction between host differentials and *F. udum* isolates, and the matrix of interaction means (11 genotypes vs. 60 isolates) were illustrated by PCA biplot [51] in R statistical program. The angles between vectors drowning for the genotypes were used to evaluate the similarity of genotype resistance or susceptibility by considering the distance of genotypes from the *F. udum* isolates in the biplot. The numeric disease reaction values were converted into characters as R-resistant (0–10%), MR-moderately resistant (10–30%) and S-susceptible (> 30%). Isolates were grouped as variants based on the reaction of each isolate against host differentials and distribution maps were created (Fig. 3) by Tableau software (2019.2).

## Supplementary Information

**Additional file 1.**

## Data Availability

The data and materials that support the findings of this study are available from the corresponding author upon reasonable request.

## Abbreviations

GPS: Global positioning system
PDA: Potato Dextrose Agar
ITS: Internal transcribed spacer
PCR: Polymerase chain reaction
