# Supplementary material for: Distribution and pathogenic diversity in Fusarium udum Butler isolates: the causal agent of pigeonpea Fusarium wilt
Source: BMC Plant Biol. 2022 Mar 26;22:147. doi: 10.1186/s12870-022-03526-8 (PMC8962003; doi:10.1186/s12870-022-03526-8)
Supplement: Supplementary file 1 — Additional file 1. [file 12870_2022_3526_MOESM1_ESM.docx]

**Supplementary table 1a** Prevalence of pigeonpea Fusarium wilt incidence in served location of India

| **Year** | **State** | **District** | **Taluk** | **Village** | **Cultivar** | **Soil Type** | **Per cent wilt** | **Average wilt (%)** |
| --- | --- | --- | --- | --- | --- | --- | --- | --- |
| 2013 | Karnataka | Raichur | Raichur | Hunasalahuda | Local | Vertisol | 12 | 11.72 |
| 2013 | Karnataka | Raichur | Raichur | Muranapura | TS 3R | Vertisol | 0 |  |
| 2013 | Karnataka | Raichur | Raichur | Kalmala | Local | Vertisol | 10.33 |  |
| 2013 | Karnataka | Raichur | Manvi | Shakapur | Local | Vertisol | 22.33 |  |
| 2013 | Karnataka | Raichur | Manvi | Siravara | Local | Vertisol | 13.33 |  |
| 2013 | Karnataka | Raichur | Manvi | Jakkaladinni | BSMR-736 | Vertisol | 4 |  |
| 2013 | Karnataka | Raichur | Deodurga | Chikka Bidiru | TS- 3R | Vertisol | 6 |  |
| 2013 | Karnataka | Raichur | Deodurga | Deodurga | Local | Vertisol | 19 |  |
| 2013 | Karnataka | Raichur | Deodurga | Halladevar Gudda | BSMR-736 | Vertisol | 2.8 |  |
| 2013 | Karnataka | Raichur | Deodurga | Matha Halli | Bennur local | Vertisol | 23.67 |  |
| 2013 | Karnataka | Yadgir | Shahapur | Madrike | Katti bheeja | Vertisol | 35 |  |
| 2013 | Karnataka | Yadgir | Shahapur | B’Gudi | Local | Vertisol | 8.33 |  |
| 2013 | Karnataka | Yadgir | Shahapur | Mudugal | Local | Alfisol | 9.33 |  |
| 2013 | Karnataka | Yadgir | Shahapur | Hoskera | Karitogari | Alfisol | 14.92 |  |
| 2013 | Karnataka | Yadgir | Shahapur | Gundalli Tanda | Maruthi | Vertisol | 2.67 |  |
| 2013 | Karnataka | Yadgir | Shahapur | Gogi | TS- 3R | Vertisol | 0 |  |
| 2013 | Karnataka | Yadgir | Shorapur | Bhyrimaradi | Asha | Alfisol | 1.73 |  |
| 2013 | Karnataka | Yadgir | Shorapur | Laxmipur | TS- 3R | Alfisol | 0 |  |
| 2013 | Karnataka | Yadgir | Shorapur | Krishnapur | ICPL 87 | Alfisol | 7 |  |
| 2013 | Karnataka | Kalaburagi | Kalaburagi | ARS,Kalaburagi | TS- 3R | Vertisol | 2.9 |  |
| 2013 | Karnataka | Kalaburagi | Kalaburagi | Shirasigi | TS- 3R | Vertisol | 0 |  |
| 2013 | Karnataka | Kalaburagi | Kalaburagi | Beemahalli | Local | Vertisol | 18.82 |  |
| 2013 | Karnataka | Kalaburagi | Kalaburagi | Hirapur | BSMR-736 | Alfisol | 0 |  |
| 2013 | Karnataka | Kalaburagi | Aland | Kadaganchi | Bennur local | Vertisol | 15.6 |  |
| 2013 | Karnataka | Kalaburagi | Aland | Telkarni | Karitogari | Vertisol | 24.54 |  |
| 2013 | Karnataka | Kalaburagi | Aland | Honnalli | Karitogari | Alfisol | 36.29 |  |
| 2013 | Karnataka | Kalaburagi | Aland | Padavasalli | Local | Alfisol | 13.59 |  |
| 2013 | Karnataka | Kalaburagi | Chittapur | Diggao | Maruthi | Vertisol | 11.25 |  |
| 2013 | Karnataka | Kalaburagi | Chittapur | Dandoti | Gulyal red | Vertisol | 24.23 |  |
| 2013 | Karnataka | Kalaburagi | Chittapur | Halakatti | Local | Alfisol | 9.34 |  |
| 2013 | Karnataka | Kalaburagi | Sedam | Kodla | Karitogari | Vertisol | 19.22 |  |
| 2013 | Karnataka | Kalaburagi | Sedam | Adaki | Maruthi | Vertisol | 1.22 |  |
| 2013 | Karnataka | Kalaburagi | Sedam | Neelhalli | Local | Vertisol | 7.22 |  |
| 2013 | Karnataka | Bidar | Bidar | Honnadi | Asha | Alfisol | 5.71 |  |
| 2013 | Karnataka | Bidar | Bidar | Bynaha | C- 11 | Vertisol | 6.67 |  |
| 2013 | Karnataka | Bidar | Bidar | Mirjapur | Local | Alfisol | 11.34 |  |
| 2013 | Karnataka | Bidar | Bidar | Janawada | BSMR-736 | Vertisol | 11.26 |  |
| 2013 | Karnataka | Bidar | Basava-kalyan | Tadola | Maruthi | Alfisol | 5 |  |
| 2013 | Karnataka | Bidar | Basava-kalyan | Hipparaga | Local | Alfisol | 0 |  |
| 2013 | Karnataka | Bidar | Basava-kalyan | Manavalli | Maruthi | Alfisol | 0 |  |
| 2013 | Karnataka | Bidar | Humnabad | Hudagi | Gulyal red | Vertisol | 16 |  |
| 2013 | Karnataka | Bidar | Humnabad | Nandagao | Asha | Vertisol | 0.67 |  |
| 2013 | Karnataka | Bidar | Humnabad | Kanakatta | Local | Alfisol | 11.66 |  |
| 2013 | Karnataka | Bidar | Humnabad | Hankuni | BSMR 853 | Alfisol | 6.34 |  |
| 2014 | Karnataka | Raichur | Raichur | Haskihala | Local | Vertisol | 24.67 |  |
| 2014 | Karnataka | Raichur | Raichur | Muranapura | BSMR-736 | Vertisol | 3.67 |  |
| 2014 | Karnataka | Raichur | Raichur | Sulthanpura | TS- 3R | Alfisol | 2.33 |  |
| 2014 | Karnataka | Raichur | Manvi | Neelgal | Bennur local | Vertisol | 9.33 |  |
| 2014 | Karnataka | Raichur | Manvi | Kallur | TS- 3R | Vertisol | 0 |  |
| 2014 | Karnataka | Raichur | Manvi | Kallur | Asha | Vertisol | 0 |  |
| 2014 | Karnataka | Raichur | Deodurga | Chikkavankuni | TS- 3R | Vertisol | 0 |  |
| 2014 | Karnataka | Raichur | Deodurga | Shasaragere | Maruthi | Vertisol | 6.34 |  |
| 2014 | Karnataka | Raichur | Deodurga | Kyadegara Doddi | Local | Alfisol | 4.67 |  |
| 2014 | Karnataka | Raichur | Deodurga | Karigudda | Maruthi | Alfisol | 5.67 |  |
| 2014 | Karnataka | Raichur | Deodurga | Navilugudda | Karitogari | Vertisol | 7.34 |  |
| 2014 | Karnataka | Yadgir | Shahapur | Beeranooru | Gulyal red | Vertisol | 8.67 |  |
| 2014 | Karnataka | Yadgir | Shahapur | Hoskera | BSMR-736 | Alfisol | 6.67 |  |
| 2014 | Karnataka | Yadgir | Shahapur | Bangla Tanda | Local | Alfisol | 23.67 |  |
| 2014 | Karnataka | Yadgir | Shahapur | Gogi | BSMR-175 | Alfisol | 28.33 |  |
| 2014 | Karnataka | Yadgir | Shahapur | B. Gudi | Asha | Vertisol | 1.33 |  |
| 2014 | Karnataka | Yadgir | Shahapur | Bevinahalli | Local | Alfisol | 27 |  |
| 2014 | Karnataka | Yadgir | Shahapur | Gundalli Tanda | Gulyal local | Alfisol | 14.33 |  |
| 2014 | Karnataka | Yadgir | Shorapur | Laxmipur | Local | Vertisol | 10.67 |  |
| 2014 | Karnataka | Yadgir | Shorapur | Bhyrimaridi | Local | Vertisol | 65 |  |
| 2014 | Karnataka | Yadgir | Yadgir | Gurusunagi cross | TS- 3R | Vertisol | 2 |  |
| 2014 | Karnataka | Kalaburagi | Kalaburagi | ARS, Kalaburagi | TS-3R | Vertisol | 2.33 |  |
| 2014 | Karnataka | Kalaburagi | Kalaburagi | Daryanayak Tanda | Maruthi | Vertisol | 3.33 |  |
| 2014 | Karnataka | Kalaburagi | Kalaburagi | Pala | BSMR-736 | Vertisol | 8.67 |  |
| 2014 | Karnataka | Kalaburagi | Kalaburagi | Sannur | Local | Vertisal | 17.33 |  |
| 2014 | Karnataka | Kalaburagi | Chittapur | Vaccha | Kattibheeja | Vertisol | 53 |  |
| 2014 | Karnataka | Kalaburagi | Sedam | Evani | Bennur local | Vertisol | 63.67 |  |
| 2014 | Karnataka | Kalaburagi | Sedam | Tengli | Maruthi | Vertisol | 4.33 |  |
| 2014 | Karnataka | Kalaburagi | Sedam | Tengli cross | Bennur local | Vertisol | 31 |  |
| 2014 | Karnataka | Kalaburagi | Sedam | Huda (K) | TS- 3R | Alfisol | 3 |  |
| 2014 | Karnataka | Kalaburagi | Sedam | Shetty huda | Maruthi | Vertisol | 9.33 |  |
| 2014 | Karnataka | Kalaburagi | Sedam | Neelalli | Kattibheeja | Vertisol | 5.67 |  |
| 2014 | Karnataka | Kalaburagi | Sedam | Bheeranahalli | TS- 3R | Vertisol | 3 |  |
| 2014 | Karnataka | Bidar | Bidar | Kaplapur | BSMR-736 | Alfisol | 0 |  |
| 2014 | Karnataka | Bidar | Bidar | Dhanooru | Maruthi | Vertisol | 0 |  |
| 2014 | Karnataka | Bidar | Bhalki | Halberga | BSMR-175 | Vertisol | 17 |  |
| 2014 | Karnataka | Bidar | Bhalki | Kona- Melakunda | Local | Vertisol | 24 |  |
| 2014 | Karnataka | Bidar | Bhalki | Dharwadi | TS- 3R | Vertisol | 0 |  |
| 2014 | Karnataka | Bidar | Bhalki | Kalwadi | Local | Vertisol | 9 |  |
| 2014 | Karnataka | Bidar | Bhalki | Haranala | Maruthi | Vertisol | 16 |  |
| 2014 | Karnataka | Bidar | Humnabad | Jalasangi | Gulyal red | Alfisol | 6.33 |  |
| 2014 | Karnataka | Bidar | Humnabad | Hudagi | Local | Alfisol | 42.67 |  |
| 2014 | Karnataka | Bidar | Humnabad | Mangalagi Wadi | Maruthi | Vertisol | 0 |  |
| 2014 | Karnataka | Bidar | Humnabad | Manna- he-kelli | Asha | Vertisol | 9 |  |
| 2013 | Madhya Pradesh | Chhindawada | Chhindawada | Sanakhar | Jagrati | Vertisol | 15 | 7.41 |
| 2013 | Madhya Pradesh | Chhindawada | Chhindawada | Lonia -Maru | Jagrati | Vertisol | 10.33 |  |
| 2013 | Madhya Pradesh | Chhindawada | Chhindawada | UmariyaIsaora | Asha | Vertisol | 0.96 |  |
| 2013 | Madhya Pradesh | Chhindawada | Chourai | Dongaria | Local | Vertisol | 8.33 |  |
| 2013 | Madhya Pradesh | Chhindawada | Chourai | Khorikurd | Local | Vertisol | 19 |  |
| 2013 | Madhya Pradesh | Chhindawada | Chourai | Udaduan | Local | Vertisol | 12 |  |
| 2013 | Madhya Pradesh | Chhindawada | Chourai | Khowka | Local | Vertisol | 3.92 |  |
| 2013 | Madhya Pradesh | Chhindawada | Chourai | Jhilmili | Local | Alfisol | 5.92 |  |
| 2013 | Madhya Pradesh | Houshangabad | Piperya | Missra | Local | Alfisol | 8.33 |  |
| 2013 | Madhya Pradesh | Houshangabad | Piperya | Podi | Jagrati | Alfisol | 5 |  |
| 2013 | Madhya Pradesh | Houshangabad | Bankhedi | Paliyapipariya | Local | Vertisol | 2.62 |  |
| 2013 | Madhya Pradesh | Houshangabad | Bankhedi | Ganeshdham bachavani | Local | Vertisol | 4 |  |
| 2013 | Madhya Pradesh | Houshangabad | Bankhedi | Malanwara | Local | Alfisol | 1.33 |  |
| 2013 | Madhya Pradesh | Houshangabad | Babai | Budahawala | Local | Vertisol | 13.1 |  |
| 2013 | Madhya Pradesh | Houshangabad | Babai | Bamhori | Local | Alfisol | 3.27 |  |
| 2013 | Madhya Pradesh | Houshangabad | Babai | Bularia | Local | Vertisol | 1.06 |  |
| 2013 | Madhya Pradesh | Houshangabad | Shohagpur | Laanga | Asha | Vertisol | 2.62 |  |
| 2013 | Madhya Pradesh | Houshangabad | Shohagpur | Bareli | Asha | Alfisol | 0 |  |
| 2013 | Madhya Pradesh | Narashingpur | Gadawara | Barangh | Jagrati | Vertisol | 6.33 |  |
| 2013 | Madhya Pradesh | Narashingpur | Gadawara | Nandner | Local | Vertisol | 1.33 |  |
| 2013 | Madhya Pradesh | Narashingpur | Gadawara | Pude | Local | Vertisol | 2.67 |  |
| 2013 | Madhya Pradesh | Narashingpur | Gadawara | Baalpani | Asha | Alfisol | 10.23 |  |
| 2013 | Madhya Pradesh | Narashingpur | Gadawara | Salichowk | Local | Vertisol | 6.33 |  |
| 2013 | Madhya Pradesh | Narashingpur | Narashingpur | Dolaware | Jawahar | Vertisol | 17 |  |
| 2013 | Madhya Pradesh | Narashingpur | Narashingpur | Bakhori | Local | Alfisol | 6.67 |  |
| 2013 | Madhya Pradesh | Narashingpur | Narashingpur | Mungvani | Asha | Vertisol | 3.4 |  |
| 2013 | Madhya Pradesh | Narashingpur | Narashingpur | Danghiana | Jagrati | Vertisol | 11.67 |  |
| 2013 | Madhya Pradesh | Seoni | Seoni | Karirat | Jagrati | Vertisol | 9.33 |  |
| 2013 | Madhya Pradesh | Seoni | Seoni | Seoni | Local | Vertisol | 2.33 |  |
| 2013 | Madhya Pradesh | Seoni | Seoni | Rayawada | No.148 | Vertisol | 8.33 |  |
| 2013 | Madhya Pradesh | Seoni | Chepera | Soundar Nagar | Asha | Alfisol | 1.67 |  |
| 2013 | Madhya Pradesh | Seoni | Chepera | Aronia | Jawahar | Alfisol | 19.27 |  |
| 2013 | Madhya Pradesh | Seoni | Chepera | Devgauv | Local | Vertisol | 9.67 |  |
| 2013 | Madhya Pradesh | Seoni | Lakhnadon | Guyya | Jawahar | Vertisol | 32.55 |  |
| 2013 | Madhya Pradesh | Seoni | Lakhnadon | Sirmangni | Asha | Alfisol | 0 |  |
| 2014 | Madhya Pradesh | Chhindawada | Chourai | Khowka | Asha | Vertisol | 0 |  |
| 2014 | Madhya Pradesh | Chhindawada | Chourai | Samaswara | Asha | Vertisol | 5.33 |  |
| 2014 | Madhya Pradesh | Chhindawada | Chourai | Dongaria | Jagrathi | Alfisol | 8.33 |  |
| 2014 | Madhya Pradesh | Chhindawada | Chourai | Chourai | Local | Vertisol | 6.33 |  |
| 2014 | Madhya Pradesh | Chhindawada | Chourai | Naveguav | Local | Vertisol | 6.67 |  |
| 2014 | Madhya Pradesh | Chhindawada | Chourai | Udaduan | Jagrathi | Vertisol | 22.67 |  |
| 2014 | Madhya Pradesh | Chhindawada | Chourai | Markhadi | JA-4 | Vertisol | 16.34 |  |
| 2014 | Madhya Pradesh | Chhindawada | Chourai | Jhilmili | Asha | Vertisol | 0 |  |
| 2014 | Madhya Pradesh | Chhindawada | Chhindawada | Lonia- maru | Local | Vertisol | 14.67 |  |
| 2014 | Madhya Pradesh | Chhindawada | Chhindawada | Umariya Isaora | Local | Vertisol | 15.33 |  |
| 2014 | Madhya Pradesh | Houshangabad | Babai | Budhawala | Local | Vertisol | 0 |  |
| 2014 | Madhya Pradesh | Houshangabad | Babai | Bularia | Jagrathi | Vertisol | 7.67 |  |
| 2014 | Madhya Pradesh | Houshangabad | Babai | Bamhori | Asha | Vertisol | 0 |  |
| 2014 | Madhya Pradesh | Houshangabad | Shohagpur | Shemriharchand | Local | Vertisol | 8 |  |
| 2014 | Madhya Pradesh | Houshangabad | Shohagpur | Laanga | Local | Alfisol | 10.33 |  |
| 2014 | Madhya Pradesh | Houshangabad | Shohagpur | Shukri | Local | Vertisol | 17 |  |
| 2014 | Madhya Pradesh | Houshangabad | Shohagpur | Bareli | Jagrathi | Vertisol | 4.33 |  |
| 2014 | Madhya Pradesh | Houshangabad | Piperiya | Rajula | Local | Alfisol | 2.33 |  |
| 2014 | Madhya Pradesh | Houshangabad | Piperiya | Rampur | Local | Alfisol | 9.33 |  |
| 2014 | Madhya Pradesh | Houshangabad | Bankhedi | Paliyapipariya | Khargoan-7 | Vertisol | 5.67 |  |
| 2014 | Madhya Pradesh | Narashingpur | Gadawara | Shalicowk | Local | Alfisol | 5 |  |
| 2014 | Madhya Pradesh | Narashingpur | Gadawara | Balkhedi | Local | Vertisol | 3.33 |  |
| 2014 | Madhya Pradesh | Narashingpur | Gadawara | Jajhenkheda | Local | Vertisol | 7.33 |  |
| 2014 | Madhya Pradesh | Narashingpur | Gadawara | Gadawara | Asha | Vertisol | 3.67 |  |
| 2014 | Madhya Pradesh | Narashingpur | Gadawara | Kondiya | Local | Vertisol | 8 |  |
| 2014 | Madhya Pradesh | Narashingpur | Gadawara | Gadawara | Asha | Vertisol | 0 |  |
| 2014 | Madhya Pradesh | Narashingpur | Narashingpur | Danghiana | Local | Vertisol | 6.67 |  |
| 2014 | Madhya Pradesh | Narashingpur | Narashingpur | Baal Pani | Jagrathi | Vertisol | 8.67 |  |
| 2014 | Madhya Pradesh | Narashingpur | Narashingpur | Devnagar | Local | Vertisol | 8.33 |  |
| 2014 | Madhya Pradesh | Narashingpur | Narashingpur | Mungvaani | JA-4 | Alfisol | 9.33 |  |
| 2014 | Madhya Pradesh | Narashingpur | Narashingpur | Dhobi | Asha | Vertisol | 0 |  |
| 2014 | Madhya Pradesh | Seoni | Lakhnadon | Gorabibi | Local | Vertisol | 31 |  |
| 2014 | Madhya Pradesh | Seoni | Lakhnadon | Parasiya | Local | Alfisol | 1.67 |  |
| 2014 | Madhya Pradesh | Seoni | Lakhnadon | Sirmangni | Jagrati | Vertisol | 15.33 |  |
| 2014 | Madhya Pradesh | Seoni | Lakhnadon | Bamhori | Asha | Vertisol | 1 |  |
| 2014 | Madhya Pradesh | Seoni | Lakhnadon | Guyya | Local | Alfisol | 13.67 |  |
| 2014 | Madhya Pradesh | Seoni | Lakhnadon | Ghunai | Local | Alfisol | 0.67 |  |
| 2014 | Madhya Pradesh | Seoni | Chepera | Randheera Nagar | Asha | Alfisol | 3.55 |  |
| 2014 | Madhya Pradesh | Seoni | Chepera | Aronia | Local | Vertisol | 2.67 |  |
| 2014 | Madhya Pradesh | Seoni | Seoni | Seoni | Arhar-4 | Vertisol | 7.67 |  |
| 2014 | Madhya Pradesh | Seoni | Seoni | Seoni | Asha | Vertisol | 0 |  |
| 2013 | Maharashtra | Latur | Udgiri | Lohara | Asha | Vertisol | 0 | 9.88 |
| 2013 | Maharashtra | Latur | Udgiri | Narsigavari | Maruthi | Vertisol | 0 |  |
| 2013 | Maharashtra | Latur | Udgiri | Valandi | Maruthi | Vertisol | 42.67 |  |
| 2013 | Maharashtra | Latur | Udgiri | Dolagaon | Local | Vertisol | 19.85 |  |
| 2013 | Maharashtra | Latur | Latur | Boravati | Maruthi | Vertisol | 8.56 |  |
| 2013 | Maharashtra | Latur | Latur | Nehru Nagar | BSMR-736 | Alfisol | 7.4 |  |
| 2013 | Maharashtra | Latur | Renapur | Kudwa Tanda | Local | Vertisol | 9.14 |  |
| 2013 | Maharashtra | Latur | Renapur | Mahapur | BDN-2 | Vertisol | 5.17 |  |
| 2013 | Maharashtra | Latur | Renapur | Morwada | BDN-7 | Vertisol | 7.05 |  |
| 2013 | Maharashtra | Latur | Renapur | Kumari | Local | Alfisol | 9.62 |  |
| 2013 | Maharashtra | Parbhani | Manavat | Rudhi | Maruthi | Vertisol | 13.25 |  |
| 2013 | Maharashtra | Parbhani | Manavat | Ratnapur | Maruthi | Vertisol | 7.78 |  |
| 2013 | Maharashtra | Parbhani | Parbhani | Dharmapur | Asha | Vertisol | 0 |  |
| 2013 | Maharashtra | Parbhani | Parbhani | Parbhani | BDN-2 | Vertisol | 4.76 |  |
| 2013 | Maharashtra | Parbhani | Parbhani | Kolha | Asha | Alfisol | 0 |  |
| 2013 | Maharashtra | Parbhani | Parbhani | Jhari | BDN-2 | Vertisol | 0 |  |
| 2013 | Maharashtra | Parbhani | Parbhani | Pedgaon | Maruthi | Vertisol | 6.08 |  |
| 2013 | Maharashtra | Parbhani | Jintoor | Bhuri | Local | Alfisol | 18.65 |  |
| 2013 | Maharashtra | Parbhani | Jintoor | Jintoor | BDN-7 | Alfisol | 20.54 |  |
| 2013 | Maharashtra | Parbhani | Jintoor | Malegaon | Local | Alfisol | 17.17 |  |
| 2013 | Maharashtra | Akola | Patoor | Patoor | Asha | Vertisol | 0 |  |
| 2013 | Maharashtra | Akola | Balamau | Nauminlakharwala | Maruthi | Alfisol | 3.67 |  |
| 2013 | Maharashtra | Akola | Akola | PRC-PDKV Akola | BDN-1 | Vertisol | 2.9 |  |
| 2013 | Maharashtra | Akola | Akola | Borgaon Maju | BDN-2 | Vertisol | 0 |  |
| 2013 | Maharashtra | Akola | Akola | Vani Rambhapur | Local | Alfisol | 0 |  |
| 2013 | Maharashtra | Akola | Murtizapur | Amrora | Local | Alfisol | 16.38 |  |
| 2013 | Maharashtra | Akola | Murtizapur | Kharb | BSMR-853 | Vertisol | 22.98 |  |
| 2013 | Maharashtra | Akola | Murtizapur | Kurum | Maruthi | Vertisol | 0 |  |
| 2013 | Maharashtra | Solapur | Akkalkote | Karjal | Gulyal Red and TS 3R | Vertisol | 15 |  |
| 2013 | Maharashtra | Solapur | Akkalkote | Konalli | Kattibheeja | Vertisol | 26.33 |  |
| 2013 | Maharashtra | Solapur | Akkalkote | Dahitnawadi | Local | Vertisol | 10.67 |  |
| 2013 | Maharashtra | Solapur | Akkalkote | Byagalli | Maruthi | Vertisol | 2.19 |  |
| 2013 | Maharashtra | Solapur | Solapur | Kamti | TS 3R | Vertisol | 0 |  |
| 2013 | Maharashtra | Solapur | Solapur | Shingoli | Gulyal Red | Vertisol | 13 |  |
| 2013 | Maharashtra | Solapur | Solapur | Shingoli - 2 | TS 3R | Vertisol | 0 |  |
| 2013 | Maharashtra | Solapur | Solapur | Limbichincholi | Karitogari | Vertisol | 34 |  |
| 2013 | Maharashtra | Solapur | Solapur | Dhevgauv | BSMR-736 | Vertisol | 6 |  |
| 2013 | Maharashtra | Solapur | Solapur | Togralli | Local | Alfisol | 24.13 |  |
| 2014 | Maharashtra | Latur | Latur | Nehru nagar | Local | Vertisol | 26 |  |
| 2014 | Maharashtra | Latur | Latur | Boravati | Gulyal red | Vertisol | 24.33 |  |
| 2014 | Maharashtra | Latur | Renapur | Bardhapur | Local | Vertisol | 7 |  |
| 2014 | Maharashtra | Latur | Renapur | Morwada | BDN-2 | Vertisol | 3.33 |  |
| 2014 | Maharashtra | Latur | Renapur | Rakhmapur | Local | Vertisol | 12 |  |
| 2014 | Maharashtra | Latur | Renapur | Khanapur | BDN-7 | Vertisol | 5.33 |  |
| 2014 | Maharashtra | Latur | Renapur | Mahapur | BDN-2 | Vertisol | 4.33 |  |
| 2014 | Maharashtra | Latur | Renapur | Kudwa Tanda | Maruthi | Vertisol | 16.8 |  |
| 2014 | Maharashtra | Latur | Udgiri | Valandi | BDN-1 | Vertisol | 4.29 |  |
| 2014 | Maharashtra | Parbhani | Parbhani | Parbhani local | BDN- 2 | Vertisol | 1 |  |
| 2014 | Maharashtra | Parbhani | Parbhani | Pedgaon | Local | Vertisol | 17.33 |  |
| 2014 | Maharashtra | Parbhani | Parbhani | Kolha | Local | Vertisol | 15.66 |  |
| 2014 | Maharashtra | Parbhani | Manavat | Manavat road station | BDN-2 | Vertisol | 0 |  |
| 2014 | Maharashtra | Parbhani | Manavat | Rudhi | BDN-2 | Alfisol | 5 |  |
| 2014 | Maharashtra | Parbhani | Manavat | Ratnapur | Local | Vertisol | 32.33 |  |
| 2014 | Maharashtra | Parbhani | Pathri | Pathri | TS- 3R | Vertisol | 3.33 |  |
| 2014 | Maharashtra | Parbhani | Pathri | Pohatakli | Local | Alfisol | 34.67 |  |
| 2014 | Maharashtra | Parbhani | Pathri | Kekarjwala | Maruthi | Vertisol | 7.67 |  |
| 2014 | Maharashtra | Parbhani | Pathri | Waghala | Local | Vertisol | 49.67 |  |
| 2014 | Maharashtra | Parbhani | Sonpeth | Vita | BDN-2 | Vertisol | 1.33 |  |
| 2014 | Maharashtra | Parbhani |  | Vani sangam | BDN-2 | Vertisol | 2.33 |  |
| 2014 | Maharashtra | Akola | Akola | PRC-PDKV campus | Maruthi | Vertisol | 0 |  |
| 2014 | Maharashtra | Akola | Akola | Dongargaon | Asha | Vertisol | 0 |  |
| 2014 | Maharashtra | Akola | Akola | Boragoan maju | BSMR736 | Vertisol | 1.23 |  |
| 2014 | Maharashtra | Akola | Murtizapur | Murtizapur | BDN-7 | Vertisol | 0 |  |
| 2014 | Maharashtra | Akola | Murtizapur | Amrora | BDN-1 | Vertisol | 2.33 |  |
| 2014 | Maharashtra | Akola | Murtizapur | Kurum | Asha | Alfisol | 0 |  |
| 2014 | Maharashtra | Akola | Murtizapur | Kharb | BDN-1 | Alfisol | 0.25 |  |
| 2014 | Maharashtra | Akola | Balamau | Nauminlakharwala | Maruthi | Vertisol | 1.92 |  |
| 2014 | Maharashtra | Solapur | Akkalkote | Karjal | Maruthi | Alfisol | 7.33 |  |
| 2014 | Maharashtra | Solapur | Akkalkote | Konalli | Local | Vertisol | 12.33 |  |
| 2014 | Maharashtra | Solapur | Akkalkote | Byagalli | Karitogari | Vertisol | 7 |  |
| 2014 | Maharashtra | Solapur | Akkalkote | Akkalkote | Maruthi | Vertisol | 0 |  |
| 2014 | Maharashtra | Solapur | Akkalkote | Hasapura | Local | Vertisol | 9.33 |  |
| 2014 | Maharashtra | Solapur | Solapur | Kumbahari | Local | Vertisol | 14 |  |
| 2014 | Maharashtra | Solapur | Solapur | Valasang | Karitogari | Vertisol | 7.33 |  |
| 2014 | Maharashtra | Solapur | Solapur | Thilyal | Maruthi | Alfisol | 7.67 |  |
| 2014 | Maharashtra | Solapur | Solapur | Limbi Chincholi | local | Vertisol | 16.67 |  |
| 2014 | Maharashtra | Solapur | Solapur | Togralli | Mahabheeja | Vertisol | 12.67 |  |
| 2014 | Maharashtra | Solapur | Solapur | Togralli | Local | Vertisol | 22.33 |  |
| 2013 | Tamil Nadu | Krishnagiri | Hosur | Doddooru | Local | Alfisol | 7.67 | 6.87 |
| 2013 | Tamil Nadu | Krishnagiri | Hosur | Bheemanayak Palli | Local | Alfisol | 5 |  |
| 2013 | Tamil Nadu | Krishnagiri | Hosur | Sundagiri | Asha | Alfisol | 0 |  |
| 2013 | Tamil Nadu | Krishnagiri | Krishnagiri | Sundampatti | Local | Alfisol | 32.33 |  |
| 2013 | Tamil Nadu | Krishnagiri | Krishnagiri | Kandikuppam | Vamban | Alfisol | 11 |  |
| 2013 | Tamil Nadu | Krishnagiri | Krishnagiri | Peripuliarasai | Asha | Alfisol | 0 |  |
| 2013 | Tamil Nadu | Krishnagiri | Vadagalabatti | Undupatti | Local | Vertisol | 4.33 |  |
| 2013 | Tamil Nadu | Krishnagiri | Uttangarai | Kodumanda | Local | Vertisol | 3.53 |  |
| 2013 | Tamil Nadu | Krishnagiri | Uttangarai | patti |  |  |  |  |
| 2013 | Tamil Nadu | Krishnagiri | Uttangarai | Sambal patti | Local | Alfisol | 19.26 |  |
| 2013 | Tamil Nadu | Dharmapuri | Palakodu | Perayambetti gate | Local | Alfisol | 0 |  |
| 2013 | Tamil Nadu | Dharmapuri | Palakodu | Periyambatti | Local | Alfisol | 5.67 |  |
| 2013 | Tamil Nadu | Dharmapuri | Palakodu | Kaarimangalam | Vamban | Alfisol | 11.25 |  |
| 2013 | Tamil Nadu | Dharmapuri | Palakodu | Baisalyae | Local | Alfisol | 3.33 |  |
| 2013 | Tamil Nadu | Dharmapuri | Dharmapuri | Motupatti | Asha | Alfisol | 0 |  |
| 2013 | Tamil Nadu | Dharmapuri | Pochampalli | Kallanoor | Local | Vertisol | 8.23 |  |
| 2013 | Tamil Nadu | Dharmapuri | Pochampalli | Kalarpatti | C-11 | Vertisol | 5.91 |  |
| 2013 | Tamil Nadu | Dharmapuri | Arure | Irumattur | Local | Vertisol | 0 |  |
| 2013 | Tamil Nadu | Thiruvenamalai | Thiruvena-malai | Ladavaram | C-11 | Alfisol | 1.33 |  |
| 2013 | Tamil Nadu | Thiruvenamalai | Thiruvena-malai | Ayyampadur | Local | Alfisol | 8.33 |  |
| 2013 | Tamil Nadu | Thiruvenamalai | Thiruvena-malai | Kariandal | Asha | Alfisol | 0 |  |
| 2013 | Tamil Nadu | Thiruvenamalai | Chatram | Aryamagalam | Local | Alfisol | 13.67 |  |
| 2013 | Tamil Nadu | Thiruvenamalai | Chatram | Kannakurki | Local | Alfisol | 6.34 |  |
| 2013 | Tamil Nadu | Thiruvenamalai | Chatram | Rolapudi | Local | Alfisol | 5.66 |  |
| 2013 | Tamil Nadu | Thiruvenamalai | Poloor | Ayyambalai | Local | Vertisol | 33.33 |  |
| 2013 | Tamil Nadu | Thiruvenamalai | Poloor | Murugapadi | Local | Alfisol | 2.33 |  |
| 2013 | Tamil Nadu | Vellore | Vellore | Backmarpet | Asha | Alfisol | 0 |  |
| 2013 | Tamil Nadu | Vellore | Vellore | Santhamadurai | CO-11 | Vertisol | 21.67 |  |
| 2013 | Tamil Nadu | Vellore | Vellore | Kaniyambadi | Local | Alfisol | 10.33 |  |
| 2013 | Tamil Nadu | Vellore | Vellore | Melvallum | Asha | Alfisol | 0.67 |  |
| 2013 | Tamil Nadu | Vellore | Thirupattor | Narayanapuram | Local | Alfisol | 6.67 |  |
| 2013 | Tamil Nadu | Vellore | Thirupattor | Rajavoor | Local | Alfisol | 14 |  |
| 2013 | Tamil Nadu | Vellore | Thirupattor | Kannalapatti | Local | Alfisol | 5 |  |
| 2013 | Tamil Nadu | Vellore | Arni | Ballam | Asha | Alfisol | 0 |  |
| 2014 | Tamil Nadu | Krishnagiri | Hosur | Gammandoddi | C-11 | Alfisol | 4 |  |
| 2014 | Tamil Nadu | Krishnagiri | Hosur | Koneripalli | Local | Alfisol | 5.67 |  |
| 2014 | Tamil Nadu | Krishnagiri | Hosur | Sundagiri | Local | Alfisol | 0 |  |
| 2014 | Tamil Nadu | Krishnagiri | Hosur | Chinnar | C-11 | Alfisol | 6 |  |
| 2014 | Tamil Nadu | Krishnagiri | Krishnagiri | Kurubarahalli | Local | Vertisol | 3.67 |  |
| 2014 | Tamil Nadu | Krishnagiri | Krishnagiri | Peripuliarasai | Asha | Vertisol | 3.33 |  |
| 2014 | Tamil Nadu | Krishnagiri | Uttangarai | Sappanipatti | CO-6 | Alfisol | 3 |  |
| 2014 | Tamil Nadu | Krishnagiri | Uttangarai | Kamala Pura | CO-6 | Alfisol | 6.67 |  |
| 2014 | Tamil Nadu | Krishnagiri | Uttangarai | Kodumanda-patti | Asha | Alfisol | 0 |  |
| 2014 | Tamil Nadu | Krishnagiri |  | Sambal patti | Local | Vertisol | 36.33 |  |
| 2014 | Tamil Nadu | Dharmapuri | Palakodu | Manikattiyar | Local | Vertisol | 10.34 |  |
| 2014 | Tamil Nadu | Dharmapuri | Palakodu | Karimangalam | Local | Alfisol | 3 |  |
| 2014 | Tamil Nadu | Dharmapuri | Palakodu | Chiyambatti | Asha | Alfisol | 4.33 |  |
| 2014 | Tamil Nadu | Dharmapuri | Palakodu | Thindal | Local | Alfisol | 11.34 |  |
| 2014 | Tamil Nadu | Dharmapuri | Palakodu | Savalu patti | Asha | Alfisol | 0 |  |
| 2014 | Tamil Nadu | Dharmapuri | Palakodu | Pethanur | Khargoan-1 | Alfisol | 9.67 |  |
| 2014 | Tamil Nadu | Dharmapuri | Arur | Irumattur | Vamban | Alfisol | 4.33 |  |
| 2014 | Tamil Nadu | Dharmapuri | Pochampalli | Kallanoor | Khargoan-1 | Alfisol | 5.33 |  |
| 2014 | Tamil Nadu | Dharmapuri | Pochampalli | Vadamala patti | Local | Alfisol | 8.33 |  |
| 2014 | Tamil Nadu | Dharmapuri | Pochampalli | Kalarpatti | Asha | Alfisol | 0 |  |
| 2014 | Tamil Nadu | Thiruvenamalai | Thiruvenamalai | Ayyapalyam pudur | Local | Alfisol | 53.67 |  |
| 2014 | Tamil Nadu | Thiruvenamalai | Thiruvenamalai | Periapolapadi | C-11 | Alfisol | 5.33 |  |
| 2014 | Tamil Nadu | Thiruvenamalai | Thiruvenamalai | Kannakurki | Local | Alfisol | 2 |  |
| 2014 | Tamil Nadu | Thiruvenamalai | Thiruvenamalai | Chatram | Asha | Vertisol | 0 |  |
| 2014 | Tamil Nadu | Thiruvenamalai | Poloor | Shanthipuram | Asha | Vertisol | 0 |  |
| 2014 | Tamil Nadu | Thiruvenamalai | Poloor | Nayadi mangalam | Local | Alfisol | 6.67 |  |
| 2014 | Tamil Nadu | Thiruvenamalai | Poloor | Backmarpet | Local | Vertisol | 4.33 |  |
| 2014 | Tamil Nadu | Thiruvenamalai | Poloor | Puliyondagla | Local | Vertisol | 2.33 |  |
| 2014 | Tamil Nadu | Thiruvenamalai | Kalsapakam | Motupaluam | Asha | Alfisol | 0 |  |
| 2014 | Tamil Nadu | Thiruvenamalai | Kalsapakam | Kuruvimalai | Local | Vertisol | 9 |  |
| 2014 | Tamil Nadu | Vellore | Arni | Palayeer | Vamban | Vertisol | 0 |  |
| 2014 | Tamil Nadu | Vellore | Arni | Vannangalam | Local | Vertisol | 3 |  |
| 2014 | Tamil Nadu | Vellore | Arni | Honnupuram | Khargoan-1 | Vertisol | 8.67 |  |
| 2014 | Tamil Nadu | Vellore | Arni | Ballam | Local | Alfisol | 5.33 |  |
| 2014 | Tamil Nadu | Vellore | Thirupattor | Sunnam Kottai | Local | Alfisol | 8.33 |  |
| 2014 | Tamil Nadu | Vellore | Vellore | Kaniyambadi | Local | Alfisol | 7.67 |  |
| 2014 | Tamil Nadu | Vellore | Vellore | Sapthalivaram | Local | Alfisol | 6.67 |  |
| 2014 | Tamil Nadu | Vellore | Vellore | Thirumalai Kodi | Asha | Alfisol | 0 |  |
| 2014 | Tamil Nadu | Vellore | Anekattu | Munayambatti | Vambhan | Vertisol | 0 |  |
| 2013 | Telangana | Warangal | Narsampet | Kamalapuram | Maruthi | Alfisol | 10.67 | 9.43 |
| 2013 | Telangana | Warangal | Narsampet | Kondasamudram | Local | Alfisol | 9 |  |
| 2013 | Telangana | Warangal | Narsampet | Ippiguda | Local | Vertisol | 14.05 |  |
| 2013 | Telangana | Warangal | Sangam | Ramnagar | Abhaya | Vertisol | 12.33 |  |
| 2013 | Telangana | Warangal | Geesugonda | Kondagiri | Asha | Vertisol | 0 |  |
| 2013 | Telangana | Warangal | Geesugonda | Shakapur | Local | Alfisol | 4.67 |  |
| 2013 | Telangana | Warangal | Geesugonda | Komala | LRG-30 | Alfisol | 11.33 |  |
| 2013 | Telangana | Warangal | Duggadi | Girnibhavi | LRG-30 | Alfisol | 9.67 |  |
| 2013 | Telangana | Medak | Sangareddy | Ismailkhanpet | Local | Vertisol | 23.67 |  |
| 2013 | Telangana | Medak | Sangareddy | Kandi | Local | Vertisol | 21 |  |
| 2013 | Telangana | Medak | Sadashivapet | Nandikandi | Local | Vertisol | 10.33 |  |
| 2013 | Telangana | Medak | Sadashivapet | Arure | LRG-30 | Alfisol | 12.34 |  |
| 2013 | Telangana | Medak | Munipalli | Bhudera | Local | Vertisol | 10.67 |  |
| 2013 | Telangana | Medak | Shankerpally | Elvarti | TS-3R | Alfisol | 6.3 |  |
| 2013 | Telangana | Medak | Raykodu | Shirur | Local | Vertisol | 12.33 |  |
| 2013 | Telangana | Medak | Alladurga | Chevella | Asha | Vertisol | 0 |  |
| 2013 | Telangana | Medak | Pulkal | Chotkur | Maruthi | Vertisol | 3.92 |  |
| 2013 | Telangana | Medak | Pulkal | Honnapur | Local | Vertisol | 9.61 |  |
| 2013 | Telangana | Rangareddy | Chevella | Gollapalle | TS-3R | Alfisol | 0 |  |
| 2013 | Telangana | Rangareddy | Chevella | Kistapur | Laxmi | Vertisol | 5.34 |  |
| 2013 | Telangana | Rangareddy | Chevella | Kowkuntla | Local | Alfisol | 0 |  |
| 2013 | Telangana | Rangareddy | Chevella | Gundal | Local | Alfisol | 15.68 |  |
| 2013 | Telangana | Rangareddy | Paroor | Ebanoor | Asha | Alfisol | 0 |  |
| 2013 | Telangana | Rangareddy | Tandur | Tandur | LRG-30 | Alfisol | 21.92 |  |
| 2013 | Telangana | Rangareddy | Tandur | Shankarareddy | Local | Alfisol | 13.34 |  |
| 2013 | Telangana | Rangareddy | Tandur | palli |  |  |  |  |
| 2013 | Telangana | Rangareddy | Tandur | Machanoor | Local | Alfisol | 6.33 |  |
| 2013 | Telangana | Rangareddy | Tandur | Malkapur | Local | Vertisol | 10.66 |  |
| 2013 | Telangana | Rangareddy | Tandur | Inole | TS- 3R | Alfisol | 0 |  |
| 2013 | Telangana | Rangareddy | Parigi | Chityal | Local | Vertisol | 3.34 |  |
| 2013 | Telangana | Rangareddy | Parigi | Narayanpur | Local | Alfisol | 0 |  |
| 2013 | Telangana | Rangareddy | Parigi | Ibrahimpur | Maruthi | Alfisol | 5.22 |  |
| 2013 | Telangana | Mahbubnagar | Pebbair | Rangapur | TS-3R | Alfisol | 0 |  |
| 2013 | Telangana | Mahbubnagar | Pebbair | Gummadam | Asha | Alfisol | 0 |  |
| 2013 | Telangana | Mahbubnagar | Pebbair | Nundavalli | LRG-30 | Alfisol | 4 |  |
| 2013 | Telangana | Mahbubnagar | Kodangal | Parsapur | LRG-30 | Vertisol | 26.34 |  |
| 2013 | Telangana | Mahbubnagar | Kodangal | Nagaram | Local | Vertisol | 5.34 |  |
| 2013 | Telangana | Mahbubnagar | Kodangal | Husanabad | Local | Alfisol | 9.67 |  |
| 2013 | Telangana | Mahbubnagar | Kodangal | Mohamadbad | Asha | Vertisol | 0 |  |
| 2013 | Telangana | Mahbubnagar | Doulatabad | Netoor | Local | Vertisol | 45.33 |  |
| 2013 | Telangana | Mahbubnagar | Doulatabad | Nandaram | ICPL 87 | Vertisol | 5 |  |
| 2013 | Telangana | Mahbubnagar | Doulatabad | Balampet | Local | Vertisol | 1.33 |  |
| 2014 | Telangana | Warangal | Duggondi | Chalparthi | TS-3R | Vertisol | 0 |  |
| 2014 | Telangana | Warangal | Duggondi | Girnibhavi | Maruthi | Vertisol | 0 |  |
| 2014 | Telangana | Warangal | Narsampet | Kamalapuram | Asha | Vertisol | 9.43 |  |
| 2014 | Telangana | Warangal | Sangam | Krishnanagar | Maruti | Vertisol | 13.77 |  |
| 2014 | Telangana | Warangal | Sangam | Ramnagar | Local | Vertisol | 0 |  |
| 2014 | Telangana | Warangal | Chityal | Ankushapur | Local | Vertisol | 14.05 |  |
| 2014 | Telangana | Warangal | Geesugonda | Kondagiri | Asha | Vertisol | 0 |  |
| 2014 | Telangana | Warangal |  | Komala | Local | Vertisol | 0 |  |
| 2014 | Telangana | Medak | Sangaraddy | Pasalwadi | LRG-30 | Alfisol | 69.33 |  |
| 2014 | Telangana | Medak | Pulkal | Honnapur | Asha | Vertisol | 0 |  |
| 2014 | Telangana | Medak | Pulkal | Chotkur | Local | Vertisol | 18.33 |  |
| 2014 | Telangana | Medak | Andol | Andol | TS-3R | Alfisol | 1 |  |
| 2014 | Telangana | Medak | Alladurg | Gadipeddapur | LRG-30 | Vertisol | 22.33 |  |
| 2014 | Telangana | Medak | Alladurg | Chilvera | Local | Vertisol | 0 |  |
| 2014 | Telangana | Medak | Alladurg | Chevella | Asha | Vertisol | 0 |  |
| 2014 | Telangana | Medak | Raykodu | Shirur | Maruthi | Vertisol | 3 |  |
| 2014 | Telangana | Medak | Raykodu | Gatpalli | Maruthi | Vertisol | 14.67 |  |
| 2014 | Telangana | Medak | Raykodu | Shirur | Local | Vertisol | 13.33 |  |
| 2014 | Telangana | Medak | Raykodu | Shirur | Maruthi | Vertisol | 6.67 |  |
| 2014 | Telangana | Medak | Naylkal | Naylkal | Local | Alfisol | 0 |  |
| 2014 | Telangana | Rangareddy | Parigi | Kankal | Local | Alfisol | 0 |  |
| 2014 | Telangana | Rangareddy | Parigi | Chityal | Asha | Vertisol | 0 |  |
| 2014 | Telangana | Rangareddy | Parigi | Gadisingapur | Local | Vertisol | 2.34 |  |
| 2014 | Telangana | Rangareddy | Parigi | Narayanpur | Maruthi | Vertisol | 11.67 |  |
| 2014 | Telangana | Rangareddy | Parigi | Ibraiumpur | Asha | Alfisol | 0 |  |
| 2014 | Telangana | Rangareddy | Darur | Endnoor | Asha | Alfisol | 0 |  |
| 2014 | Telangana | Rangareddy | Tandoor | Rampur | Local | Alfisol | 0 |  |
| 2014 | Telangana | Rangareddy | Tandoor | ARS Tandur | Asha | Alfisol | 0 |  |
| 2014 | Telangana | Rangareddy | Tandoor | Rasulpur | Local | Alfisol | 4 |  |
| 2014 | Telangana | Rangareddy | Tandoor | Khimaspally | LRG- 30 | Vertisol | 26.09 |  |
| 2014 | Telangana | Mahbubnagar | Kodangal | Parsapur | Local | Vertisol | 4.75 |  |
| 2014 | Telangana | Mahbubnagar | Kodangal | Nagaram | LRG-30 | Vertisol | 70.8 |  |
| 2014 | Telangana | Mahbubnagar | Kodangal | Husnabad | LRG-30 | Vertisol | 33.64 |  |
| 2014 | Telangana | Mahbubnagar | Kodangal | Rangareddy-pally | Asha | Vertisol | 0 |  |
| 2014 | Telangana | Mahbubnagar | Kodangal | Mohamdabad | Maruthi | Vertisol | 0 |  |
| 2014 | Telangana | Mahbubnagar | Kodangal | Narayanpuram | Local | Alfisol | 3.31 |  |
| 2014 | Telangana | Mahbubnagar | Doulatabad | Netoor | LRG-30 | Vertisol | 45.33 |  |
| 2014 | Telangana | Mahbubnagar | Doulatabad | Nandaram | Maruthi | Vetrisol | 5 |  |
| 2014 | Telangana | Mahbubnagar | Doulatabad | Balampet | Local | Vetrisol | 1.33 |  |

**Supplementary table 1b** *Fusarium udum* isolates from different pigeonpea reproducing regions in India

| **Sl. N.** | **Isolate Code** | **Location** |
| --- | --- | --- |
| 1 | Fu 2 | Mahbubnagar, Telangana |
| 2 | Fu 3 | Medak, Telangana |
| 3 | Fu 4 | Mahbubnagar, Telangana |
| 4 | Fu 5 | Mahbubnagar, Telangana |
| 5 | Fu 6 | Mahbubnagar, Telangana |
| 6 | Fu 7 | Mahbubnagar, Telangana |
| 7 | Fu 8 | Mahbubnagar, Telangana |
| 8 | Fu 9 | Medak, Telangana |
| 9 | Fu 10 | Medak, Telangana |
| 10 | Fu 11 | Medak, Telangana |
| 11 | Fu 12 | Warangal, Telangana |
| 12 | Fu 13 | Warangal, Telangana |
| 13 | Fu 14 | Warangal, Telangana |
| 14 | Fu 15 | Warangal, Telangana |
| 15 | Fu 16 | Warangal, Telangana |
| 16 | Fu 17 | Rangareddy, Telangana |
| 17 | Fu 18 | Rangareddy, Telangana |
| 18 | Fu 19 | Rangareddy, Telangana |
| 19 | Fu 20 | Mahbubnagar, Telangana |
| 20 | Fu 21 | Medak, Telangana |
| 21 | Fu 22 | Bidar, Karnataka |
| 22 | Fu 23 | Raichur, Karnataka |
| 23 | Fu 24 | Bidar, Karnataka |
| 24 | Fu 25 | Mandya, Karnataka |
| 25 | Fu 26 | Ramanagaram, Karnataka |
| 26 | Fu 27 | Bangalore, Karnataka |
| 27 | Fu 28 | Raichur, Karnataka |
| 28 | Fu 29 | Raichur, Karnataka |
| 29 | Fu 31 | Kalaburagi, Karnataka |
| 30 | Fu 32 | Kalaburagi, Karnataka |
| 31 | Fu 33 | Kalaburagi, Karnataka |
| 32 | Fu 34 | Yadgir, Karnataka |
| 33 | Fu 35 | Kalaburagi, Karnataka |
| 34 | Fu 36 | Chitradurga, Karnataka |
| 35 | Fu 37 | Kalaburagi, Karnataka |
| 36 | Fu 38 | Kalaburagi, Karnataka |
| 37 | Fu 39 | Bidar, Karnataka |
| 38 | Fu 40 | Bidar, Karnataka |
| 39 | Fu 41 | Raichur, Karnataka |
| 40 | Fu 42 | Kalaburagi, Karnataka |
| 41 | Fu 43 | Kalaburagi, Karnataka |
| 42 | Fu 44 | Raichur, Karnataka |
| 43 | Fu 45 | Yadgir, Karnataka |
| 44 | Fu 46 | Raichur, Karnataka |
| 45 | Fu 47 | Kalaburagi, Karnataka |
| 46 | Fu 48 | Kalaburagi, Karnataka |
| 47 | Fu 49 | Kalaburagi, Karnataka |
| 48 | Fu 50 | Kalaburagi, Karnataka |
| 49 | Fu 51 | Bidar, Karnataka |
| 50 | Fu 52 | Kalaburagi, Karnataka |
| 51 | Fu 53 | Yadgir, Karnataka |
| 52 | Fu 54 | Bidar, Karnataka |
| 53 | Fu 55 | Solapur, Maharashtra |
| 54 | Fu 56 | Solapur, Maharashtra |
| 55 | Fu 57 | Solapur, Maharashtra |
| 56 | Fu 58 | Jalna, Maharashtra |
| 57 | Fu 59 | Amaravati, Maharashtra |
| 58 | Fu 60 | Yavatmal, Maharashtra |
| 59 | Fu 61 | Jalna, Maharashtra |
| 60 | Fu 62 | Latur, Maharashtra |
| 61 | Fu 63 | Beed, Maharashtra |
| 62 | Fu 65 | Parbhani, Maharashtra |
| 63 | Fu 66 | Parbhani, Maharashtra |
| 64 | Fu 67 | Parbhani, Maharashtra |
| 65 | Fu 68 | Buldhana, Maharashtra |
| 66 | Fu 69 | Buldhana, Maharashtra |
| 67 | Fu 70 | Akola, Maharashtra |
| 68 | Fu 71 | Latur, Maharashtra |
| 69 | Fu 72 | Solapur, Maharashtra |
| 70 | Fu 73 | Dharmapuri, Tamil Nadu |
| 71 | Fu 74 | Vellore, Tamil Nadu |
| 72 | Fu 75 | Krishnagiri, Tamil Nadu |
| 73 | Fu 76 | Thiruvenamalai, Tamil Nadu |
| 74 | Fu 77 | Vellore, Tamil Nadu |
| 75 | Fu 78 | Coimbatore, Tamil Nadu |
| 76 | Fu 79 | Vellore, Tamil Nadu |
| 77 | Fu 80 | Vellore, Tamil Nadu |
| 78 | Fu 81 | Krishnagiri, Tamil Nadu |
| 79 | Fu 83 | Dharmapuri, Tamil Nadu |
| 80 | Fu 86 | Narashinghpur, Madhya Pradesh |
| 81 | Fu 87 | Narashinghpur, Madhya Pradesh |
| 82 | Fu 88 | Chhindawara, Madhya Pradesh |
| 83 | Fu 89 | Chhindawara, Madhya Pradesh |
| 84 | Fu 90 | Chhindawara, Madhya Pradesh |
| 85 | Fu 91 | Chhindawara, Madhya Pradesh |
| 86 | Fu 93 | Jabalpur, Madhya Pradesh |
| 87 | Fu 94 | Jabalpur, Madhya Pradesh |
| 88 | Fu 95 | Jabalpur, Madhya Pradesh |
| 89 | Fu 96 | Narashinghpur, Madhya Pradesh |
| 90 | Fu 97 | Seoni, Madhya Pradesh |
| 91 | Fu 98 | Seoni, Madhya Pradesh |
| 92 | Fu 99 | Seoni, Madhya Pradesh |
| 93 | Fu 100 | Sehore, Madhya Pradesh |
| 94 | Fu 101 | Sehore, Madhya Pradesh |
| 95 | Fu 102 | Varanasi, Uttar Pradesh |
| 96 | Fu 103 | Hissar, Haryana |
| 97 | Fu 104 | Kanpur, Uttar Pradesh |
| 98 | Fu 105 | Kanpur, Uttar Pradesh |
| 99 | Fu 106 | Kanpur, Uttar Pradesh |
| 100 | Fu 107 | IARI, New Delhi |
| 101 | Fu 108 | Bhubaneshwar, Odissa |
| 102 | Fu 109 | Kurnool, Andhra Pradesh |
| 103 | Fu 110 | Kurnool, Andhra Pradesh |
| 104 | Fu 111 | Ananthapur, Andhra Pradesh |

**Supplementary table 2** *Fusarium udum* incidence on pigeonpea host differentials under greenhouse conditions

| **Code** | **Pigeonpea host differentials (Per cent wilt incidence)** | | | | | | | | | | |
| --- | --- | --- | --- | --- | --- | --- | --- | --- | --- | --- | --- |
|  | **ICP 9174** | **BDN 2** | **Bahar** | **ICP 8863** | **C 11** | **ICP 8859** | **BDN 1** | **ICP 8858** | **ICP 8862** | **ICP 2376** | **LRG 30** |
| Fu 3 | 6.67 | 46.67 | 46.67 | 100 | 60 | 80 | 100 | 66.67 | 100 | 100 | 100 |
| Fu 4 | 6.67 | 6.67 | 13.33 | 6.67 | 46.67 | 26.67 | 53.33 | 60 | 93.33 | 93.33 | 100 |
| Fu 6 | 0 | 0 | 13.33 | 100 | 33.33 | 46.67 | 40 | 66.67 | 80 | 86.67 | 100 |
| Fu 8 | 0 | 0 | 0 | 73.33 | 26.67 | 33.33 | 93.33 | 40 | 86.67 | 86.67 | 93.33 |
| Fu 10 | 6.67 | 46.67 | 46.67 | 86.67 | 60 | 20 | 73.33 | 66.67 | 93.33 | 100 | 100 |
| Fu 11 | 6.67 | 46.67 | 46.67 | 93.33 | 46.67 | 60 | 60 | 73.33 | 100 | 86.67 | 100 |
| Fu 12 | 6.67 | 20 | 26.67 | 6.67 | 46.67 | 6.67 | 20 | 80 | 93.33 | 73.33 | 100 |
| Fu 13 | 6.67 | 13.33 | 0 | 73.33 | 20 | 40 | 53.33 | 33.33 | 80 | 60 | 73.33 |
| Fu 15 | 0 | 0 | 0 | 0 | 6.67 | 33.33 | 0 | 33.33 | 73.33 | 100 | 93.33 |
| Fu 16 | 6.67 | 6.67 | 0 | 6.67 | 6.67 | 20 | 13.33 | 13.33 | 86.67 | 73.33 | 93.33 |
| Fu 19 | 0 | 0 | 0 | 33.33 | 6.67 | 0 | 60 | 6.67 | 73.33 | 86.67 | 46.67 |
| Fu 21 | 6.67 | 6.67 | 13.33 | 53.33 | 13.33 | 40 | 33.33 | 26.67 | 86.67 | 86.67 | 80 |
| Fu 23 | 0 | 0 | 6.67 | 100 | 33.33 | 40 | 80 | 53.33 | 46.67 | 80 | 80 |
| Fu 24 | 6.67 | 6.67 | 6.67 | 86.67 | 6.67 | 93.33 | 33.33 | 6.67 | 73.33 | 100 | 93.33 |
| Fu 25 | 0 | 0 | 0 | 6.67 | 0 | 0 | 0 | 6.67 | 80 | 93.33 | 93.33 |
| Fu 27 | 0 | 0 | 0 | 53.33 | 0 | 13.33 | 0 | 33.33 | 40 | 40 | 93.33 |
| Fu 28 | 6.67 | 53.33 | 93.33 | 80 | 73.33 | 80 | 60 | 80 | 100 | 100 | 46.67 |
| Fu 29 | 6.67 | 13.33 | 0 | 0 | 40 | 26.67 | 86.67 | 33.33 | 73.33 | 73.33 | 100 |
| Fu 31 | 0 | 26.67 | 6.67 | 73.33 | 6.67 | 33.33 | 0 | 40 | 33.33 | 100 | 93.33 |
| Fu 34 | 0 | 0 | 13.33 | 40 | 6.67 | 20 | 93.33 | 33.33 | 86.67 | 100 | 100 |
| Fu 36 | 0 | 0 | 0 | 0 | 6.67 | 0 | 73.33 | 53.33 | 86.67 | 73.33 | 93.33 |
| Fu 37 | 6.67 | 20 | 26.67 | 73.33 | 6.67 | 33.33 | 53.33 | 53.33 | 80 | 100 | 100 |
| Fu 38 | 0 | 0 | 6.67 | 100 | 6.67 | 33.33 | 6.67 | 20 | 73.33 | 100 | 100 |
| Fu 42 | 6.67 | 6.67 | 26.67 | 93.33 | 20 | 80 | 86.67 | 33.33 | 86.67 | 73.33 | 100 |
| Fu 43 | 0 | 0 | 0 | 0 | 0 | 0 | 0 | 0 | 33.33 | 46.67 | 33.33 |
| Fu 46 | 0 | 0 | 6.67 | 66.67 | 6.67 | 13.33 | 0 | 33.33 | 66.67 | 100 | 93.33 |
| Fu 49 | 6.67 | 6.67 | 33.33 | 73.33 | 46.67 | 66.67 | 0 | 33.33 | 100 | 93.33 | 93.33 |
| Fu 54 | 6.67 | 40 | 40 | 86.67 | 60 | 73.33 | 100 | 86.67 | 100 | 100 | 100 |
| Fu 55 | 33.33 | 33.33 | 26.67 | 86.67 | 53.33 | 73.33 | 100 | 86.67 | 100 | 86.67 | 100 |
| Fu 58 | 0 | 6.67 | 6.67 | 46.67 | 6.67 | 33.33 | 100 | 20 | 86.67 | 93.33 | 100 |
| Fu 60 | 6.67 | 26.67 | 0 | 6.67 | 40 | 20 | 86.67 | 33.33 | 80 | 33.33 | 86.67 |
| Fu 61 | 0 | 6.67 | 0 | 100 | 66.67 | 53.33 | 100 | 40 | 100 | 100 | 100 |
| Fu 65 | 0 | 0 | 20 | 73.33 | 0 | 20 | 0 | 20 | 0 | 80 | 100 |
| Fu 68 | 6.67 | 26.67 | 33.33 | 6.67 | 60 | 73.33 | 46.67 | 86.67 | 100 | 86.67 | 100 |
| Fu 70 | 6.67 | 20 | 6.67 | 80 | 6.67 | 6.67 | 6.67 | 60 | 73.33 | 80 | 100 |
| Fu 71 | 73.33 | 46.67 | 26.67 | 100 | 53.33 | 80 | 53.33 | 53.33 | 100 | 100 | 100 |
| Fu 72 | 0 | 0 | 26.67 | 0 | 33.33 | 6.67 | 33.33 | 53.33 | 86.67 | 93.33 | 100 |
| Fu 73 | 0 | 20 | 0 | 0 | 46.67 | 20 | 46.67 | 66.67 | 86.67 | 66.67 | 93.33 |
| Fu 74 | 0 | 0 | 6.67 | 0 | 40 | 13.33 | 20 | 66.67 | 100 | 80 | 93.33 |
| Fu 75 | 0 | 13.33 | 20 | 0 | 86.67 | 20 | 66.67 | 93.33 | 100 | 80 | 93.33 |
| Fu 76 | 0 | 0 | 0 | 0 | 33.33 | 13.33 | 53.33 | 60 | 93.33 | 93.33 | 100 |
| Fu 77 | 0 | 6.67 | 26.67 | 6.67 | 66.67 | 13.33 | 20 | 93.33 | 100 | 93.33 | 100 |
| Fu 78 | 0 | 0 | 0 | 0 | 6.67 | 6.67 | 33.33 | 53.33 | 93.33 | 86.67 | 100 |
| Fu 79 | 6.67 | 6.67 | 6.67 | 93.33 | 6.67 | 20 | 0 | 53.33 | 33.33 | 100 | 100 |
| Fu 80 | 6.67 | 20 | 6.67 | 0 | 73.33 | 26.67 | 46.67 | 80 | 66.67 | 86.67 | 93.33 |
| Fu 81 | 6.67 | 13.33 | 26.67 | 93.33 | 6.67 | 33.33 | 0 | 33.33 | 33.33 | 93.33 | 100 |
| Fu 83 | 0 | 0 | 6.67 | 0 | 6.67 | 0 | 0 | 13.33 | 6.67 | 40 | 93.33 |
| Fu 86 | 0 | 0 | 20 | 6.67 | 33.33 | 26.67 | 93.33 | 60 | 33.33 | 46.67 | 100 |
| Fu 93 | 6.67 | 6.67 | 13.33 | 0 | 40 | 0 | 13.33 | 60 | 33.33 | 100 | 100 |
| Fu 95 | 6.67 | 26.67 | 20 | 0 | 33.33 | 0 | 33.33 | 46.67 | 100 | 93.33 | 100 |
| Fu 97 | 33.33 | 33.33 | 13.33 | 73.33 | 60 | 53.33 | 100 | 66.67 | 100 | 100 | 100 |
| Fu 98 | 0 | 0 | 0 | 40 | 6.67 | 6.67 | 26.67 | 6.67 | 46.67 | 66.67 | 93.33 |
| Fu 99 | 0 | 0 | 0 | 0 | 6.67 | 6.67 | 40 | 46.67 | 46.67 | 86.67 | 100 |
| Fu 100 | 0 | 0 | 0 | 80 | 6.67 | 6.67 | 73.33 | 26.67 | 53.33 | 80 | 93.33 |
| Fu 101 | 0 | 20 | 6.67 | 6.67 | 40 | 0 | 13.33 | 80 | 33.33 | 66.67 | 100 |
| Fu 103 | 0 | 0 | 0 | 33.33 | 40 | 0 | 0 | 86.67 | 33.33 | 100 | 100 |
| Fu 104 | 0 | 6.67 | 6.67 | 6.67 | 40 | 26.67 | 13.33 | 80 | 33.33 | 93.33 | 100 |
| Fu 105 | 0 | 0 | 0 | 0 | 0 | 0 | 0 | 0 | 0 | 40 | 40 |
| Fu 106 | 0 | 0 | 6.67 | 13.33 | 6.67 | 0 | 6.67 | 93.33 | 40 | 86.67 | 100 |
| Fu 107 | 6.67 | 0 | 13.33 | 6.67 | 33.33 | 13.33 | 40 | 73.33 | 100 | 86.67 | 100 |
| Average incidence | 4.48 | 10.55 | 12.24 | 37.71 | 25.87 | 25.17 | 37.91 | 4418 | 64.18 | 75.12 | 83.28 |
